# Anxiety and Performance in High‐Achieving Adolescents: Associations Among 8 General and Specific Anxiety Measures and 13 School Grades

**DOI:** 10.1002/pchj.70088

**Published:** 2026-03-12

**Authors:** Maxim Likhanov, Evgenia Alenina, Tomasz Bloniewski, Xinlin Zhou, Yulia Kovas

**Affiliations:** ^1^ Aix Marseille Univ, CNRS, CRPN Marseille France; ^2^ Affective Psychophysiology Laboratory Institute of Health Psychology, HSE University Saint Petersburg Russia; ^3^ Department of Psychology Goldsmiths University of London London UK; ^4^ Beijing Normal University Beijing China; ^5^ Hult International Business School London UK

**Keywords:** academic performance, factor analysis, general anxiety, school grades, specific anxiety

## Abstract

Despite years of research, the links between domain‐general and domain‐specific anxieties (e.g., social), as well as their links with academic performance in different domains remain poorly understood. The current study explores anxiety‐academic performance associations across eight domain‐general and domain‐specific anxiety measures (tapping into trait, state, maths, spatial, and social anxiety, worry, and anxiety sensitivity, as well as symptoms of Generalized Anxiety Disorder, GAD) and 13 school subjects in a large (*N* = ~800) sample of schoolchildren (M_age_ = 15.26), selected for high achievement in STEM, Arts, Sports, and Literature. Our data showed that all anxiety measures load onto single general anxiety factor, explaining 51% of variance; and suggesting substantive amount of unique variance in each measure. Regression analysis showed that domain‐general anxiety (e.g., trait anxiety and GAD symptoms) did not explain much variance in academic outcomes, while domain‐specific anxiety explained variance in respective domains. For example, maths anxiety was linked with Algebra and Geometry performance. The results demonstrated that the negative link between anxiety and performance is present even in adolescents with high academic achievement (i.e., adolescents with high achievement in STEM) and this link is of small‐to‐medium effect size. Interestingly, worry scale correlated positively with performance after controlling for other anxiety measures, probably reflecting this measure tapping into some motivational and/or arousal aspects of anxiety. The study provides new insights into anxiety‐performance links that can be used for further development of measures and educational interventions.

## 1 Introduction

Research has shown that anxiety is negatively associated with academic performance and can lead to the avoidance of some academic and career paths (Ahmed 2018; Cassady et al. 2019; Daker et al. 2021; Huang 2018). For example, one meta‐analysis demonstrated a negative link between maths anxiety and maths performance with an Effect Size (ES) of −0.30 (Zhang et al. 2019). Another meta‐analysis found significant associations between maths performance and maths anxiety (ES– = −0.30), general (−0.19), and test anxiety (−0.23; Caviola et al. 2022). Research also shows that verbal and spatial anxiety could contribute to not only maths performance but also to performance in other domains (Lauer et al. 2018). However, it is currently unclear to what extent these associations reflect common variance across these three types of anxiety versus the specific contribution of each anxiety to educational performance. Moreover, further research is needed into the links across different anxiety types, such as trait and state anxiety, Generalized Anxiety Disorder symptoms, anxiety sensitivity, worry, and social anxiety, as they are also shown to differentially link with achievement, are linked with each other, and are rarely investigated in relation to school achievement (Beard and Björgvinsson 2014; Doi et al. 2018). To address this gap, the current study investigates a comprehensive set of eight self‐report measures, selected to systematically capture both general (state, trait or general anxiety, symptoms of Generalized Anxiety Disorder; and worry) and domain‐specific (maths, spatial, social, and anxiety sensitivity) facets of anxiety.

### 1.1 General‐ versus Domain‐Specific Anxiety: Common Core (−s)?

Usually, anxiety is defined as an underlying feeling of worry that is not linked to specific stimuli, but rather as a general predisposition to worry (Grös et al. 2007; Hembree 1990; Spielberger 1972; Vignoli 2015; Wang et al. 2014). Such anxiety is often referred to as trait anxiety (Grös et al. 2007; Spielberger 2012; Spitzer et al. 2006). One of the most common instruments of assessing trait anxiety is the State–Trait Anxiety Inventory‐Trait (STAIT; Spielberger 1972), which requires respondents to evaluate their anxiety for the last two or more weeks, no matter what the triggers are (Elwood et al. 2012). Another term that is used to describe such anxiety is *“generalized”* anxiety (Malanchini et al. 2017), or Generalized Anxiety Disorder (GAD), measured with GAD‐7 questionnaire (Spitzer et al. 2006). GAD is associated with experiencing uncontrollable worry and is used mostly in clinical settings (Toussaint et al. 2020), with several studies applying it to non‐clinical populations (Hinz et al. 2017; Tiirikainen et al. 2019). It remains unclear whether STAIT and GAD‐7 measure the same anxiety construct or whether they tap into different constructs with partly overlapping symptoms (see Alenina et al. 2025 for some discussion and evidence from EEG research; Beard and Björgvinsson 2014; Doi et al. 2018).

Moreover, there are other instruments that tap into domain‐general anxiety but focus on a single anxiety symptom. Such domain‐general measures are positively correlated with GAD‐7 or trait anxiety (*r* = [0.50–0.70]; Dear et al. 2011; Spence et al. 2012), suggesting that they tap into a common core of ‘general anxiety’. For example, the Penn State Worry Questionnaire (PSWQ; Meyer et al. 1990), which primarily taps into worry, showed correlations ranging from 0.29 to 0.53 with several components of anxiety, including Panic/Somatic and Generalized Anxiety, but also Separation Anxiety, Social Anxiety, and School Avoidance (Gillis et al. 1995; Păsărelu et al. 2017). Furthermore, it has been suggested that worry is a common factor across mental disorders (Kertz et al. 2012; McEvoy et al. 2013). However, the moderate strength of the correlations among domain‐general measures and measures tapping into more specific aspects of anxiety (Omani‐Samani et al. 2018; Hogg et al. 2021) suggests that each of them also captures unique aspects of the construct (stable trait vs. acute symptoms, or general worry vs. more specific worry related to social situations).

In fact, several studies found weak to moderate correlations among general and specific anxiety. For example, it was shown that general anxiety moderately correlates with maths anxiety (MA)—anxiety in relation to math situations and one of the most well‐investigated domain‐specific anxieties (see Dowker et al. 2016, for review; Hill et al. 2016; Likhanov 2017; Malanchini et al. 2017; Wang et al. 2014). General anxiety was also found to correlate with social anxiety—fear and avoidance of social interactions (La Greca and Lopez 1998; Poole et al. 2017), which was also shown to correlate with worry (e.g., 0.38 between PSWQ and social anxiety in Păsărelu et al. 2017). Specific anxieties were also found to correlate with each other. For example, MA moderately correlates with spatial anxiety—the fear of performing tasks that have a spatial component, such as when asking for a way in a new city or using a map (Lawton 1994; Gibeau et al. 2023; Lauer et al. 2018; Malanchini et al. 2017). However, existing research does not allow to clarify whether specific anxieties correlate with each other because of general anxiety or some other processes common to some but not other anxiety types. For example, it is not clear whether maths and spatial anxiety overlap because they have a common core of general anxiety, because maths and spatial abilities overlap (Hawes and Ansari 2020), or because both are “activated” by social interaction (e.g., the need to provide an answer to a math teacher or ask for directions from a stranger; see, for example, a recent study that showed relatively strong correlations (0.50) between social anxiety and fear of social evaluation, and maths anxiety (Mahak et al. 2025)).

### 1.2 Overlapping but Differentially Linked to Performance

One reason why general and specific anxiety might be linked is that anxiety might include two processes: cognitive and affective (Behar et al. 2009; Wigfield and Meece 1988). Specifically, the affective component (“emotionality”) refers to nervousness and tension in testing and other academic situations and associated autonomic reactions, and the cognitive component (“worry”) reflects appraisal of one's self‐efficacy and of consequences of failure—in a specific domain and under specific circumstances (Liebert and Morris 1967). In fact, fear of anxiety manifestations is considered to be a cognitive risk factor for anxiety disorders (Ginsburg and Drake 2002). For example, the Childhood Anxiety Sensitivity Index (CASI) assesses participants' fear of different symptoms of anxiety, tapping into three factors: Physical Concerns (the fear of anxiety‐related physical sensations, such as nausea, due to beliefs these sensations will lead to physical illness); Psychological Concerns (the fear of anxiety‐related mental sensations, such as difficulty concentrating, due to beliefs that these sensations will lead to mental illness); and Social Concerns (the fear of publicly observable anxiety‐related sensations, such as shaking, due to beliefs that demonstrating anxiety will lead to social censure) (Silverman et al. 1991). Such anxiety could contribute to interactions with peers, eventually leading to social anxiety and school anxiety, which is indirectly supported by a correlation of 0.30 between error sensitivity and school anxiety; and them both belonging to the same cluster with impostor syndrome and social/school adaptation in a large network of school‐related traits (Likhanov et al. 2025).

In turn, both cognitive and emotional aspects of anxiety, in combination with negative experiences in a specific domain, might lead to the development of higher anxiety in that domain—“Domain‐specific concerns”. Indeed, research has found differential links among specific anxieties and performance in respective domains. For example, one study showed that while spatial, maths, and verbal anxiety were moderately correlated with each other, the links with performance were mostly domain‐specific (Lauer et al. 2018). Interestingly, both maths and verbal anxiety (e.g., anxiety regarding reading aloud) predicted maths performance, but the contribution of verbal anxiety disappeared when verbal ability was controlled for. Similar results were obtained for maths, test and reading anxiety in one recent study, which showed effects of maths and test anxieties, but not reading anxiety, in predicting maths performance (Sasanguie et al. 2024). Another study showed that several different anxieties, including spatial anxiety, emotional stability, state anxiety and test anxiety, independently contributed to variance in maths anxiety (Szczygieł and Hohol 2024).

This pattern of communality and specificity extends to the relationships between general and domain‐specific anxieties. A body of research confirms that general anxiety correlates moderately with specific anxieties, such as maths anxiety (Dowker et al. 2016; Hill et al. 2016), social anxiety (Baez et al. 2023), and spatial anxiety (Geer et al. 2024). Furthermore, specific anxieties correlate with each other (e.g., math and spatial anxiety; Gibeau et al. 2023; Malanchini et al. 2017). However, a critical unresolved question is whether these correlations are driven primarily by a shared, overarching general anxiety factor, or if there are additional shared processes unique to certain clusters of specific anxieties.

### 1.3 Potential Mechanisms of the Links

Recent genetically‐informative studies provided insights into the links among several general and specific anxiety measures and their links with performance. For example, one genetically informative study (Malanchini et al. 2017) found that general, maths, and spatial anxiety (navigation and rotation/visualization facets) measures were moderately correlated at both phenotypic and genetic levels. In addition, a substantial portion of genetic and non‐shared environmental influences were specific to each anxiety construct. Further, another genetically informative study explored the links between maths anxiety, maths performance, maths attitudes and general anxiety (Malanchini et al. 2020). Maths anxiety was related to maths performance and attitudes (negatively) and to general anxiety (positively). These phenotypic overlaps were largely explained by common genetic factors: for maths anxiety and maths performance and attitudes, and for maths anxiety and general anxiety. Yet another genetically‐informative study also showed that maths anxiety has independent overlaps at the genetic level with general anxiety and maths ability (Wang et al. 2014). These results point toward the need to investigate both communalities across general and specific anxiety types and their differential contributions to achievement.

In addition, specific trait anxieties may moderate state anxieties—emotional responses triggered by particular contexts (Eysenck et al. 2007; Eysenck and Calvo 1992), and are moderated by multiple factors, including personality, experiences and environments. For example, in case of maths anxiety this may create a “vicious cycle” via multiple reciprocally‐linked processes, including higher general anxiety, lower performance in maths, negative experience during maths classes, reduced motivation and aversion of maths, less practice in maths, and higher maths anxiety (Deieso and Fraser 2019; Field et al. 2019; Luttenberger et al. 2018; Wang et al. 2018).

These reciprocal links also mean that high performance in a particular domain may contribute to lower anxiety. For example, one recent study has shown in a large dataset drawn from Programme for International Student Assessment (PISA) that there is a negative link between test anxiety and school performance in maths (D'Agostino et al. 2022). Interestingly, when the analysis was conducted separately for different levels of achievement, a stronger negative association was observed among best performing students. However, such negative association between anxiety and performance in high‐performing students has not been consistently found. For example, one study found no differences between STEM‐selected and control samples in neuroticism and slightly higher general anxiety in the STEM‐selected sample (Likhanov et al. 2021). Another study did not find a link between neuroticism and academic achievement in the STEM‐selected sample but demonstrated this link in the sample of Sport‐selected adolescents (Papageorgiou et al. 2020). This suggests that these processes might differ as a function of specific anxiety and may be differentially linked with level of achievement.

### 1.4 Current Study

To sum up, research to date has not been able to clarify the links among general and specific anxieties, and performance in different domains. Most studies have investigated correlations among a limited number of specific and general anxieties (e.g., math/statistical/spatial anxieties, Gibeau et al. 2023; math and spatial anxieties, Lauer et al. 2018); focused on associations between specific anxiety and congruent performance (e.g., Barroso et al. 2021); or investigated general types of anxiety with differential outcomes (e.g., school anxiety contribution to performance in the maths and verbal domains, Likhanov et al. 2025). Moreover, research in some domains is very limited with only a few exceptions (see e.g., research in social anxiety, Vilaplana‐Pérez et al. 2021). The current study extends previous research by applying factorial and multiple regression analyses to a comprehensive set of 8 anxieties, tapping into domain‐general and domain‐specific anxieties, and featuring different components of anxiety: general/trait, state, worry, GAD symptoms, as well as in relation to specific (academic) situations, such as maths, spatial and social. Such an approach will allow to identify communalities and differences among different anxiety measures, as well as to assess whether these differences demonstrate specific links to grades in different subjects.

Moreover, the data were collected from four distinct groups of high‐achieving adolescents selected for demonstrating high results in Sciences, Arts, Literature, or Sports, enabling an exploration of how the anxiety‐achievement associations vary across domains of expertise. It can be expected that similar anxiety‐performance links will be demonstrated across all expertise groups for some anxiety measures, such as trait/state, GAD symptoms or social anxiety. In contrast, anxiety‐performance for some specific anxieties may differ as a function of expertise. For example, we can test whether maths anxiety—maths performance links differ in STEM‐selected sample compared with a non‐STEM‐selected group, exploring whether this link operates across the whole performance continuum. Given that the adolescents demonstrated high ability in different domains (i.e., differential patterns of strengths and difficulties in different domains, for example, verbal advantage in the Literature sample and maths advantage in the STEM‐selected sample), they could serve as control samples for each other.

Thus, the current paper addresses 3 main aims:

Aim 1: to quantify the pattern of correlations and test competing factorial models (a one‐factor model vs. a two‐factor ‘general‐specific’ model) for the eight anxiety measures.

Aim 2: to assess the unique predictive value of each anxiety measure for grades in 13 school subjects using multiple regression, testing for both domain‐specific and cross‐domain effects.

Aim 3: to explore whether the patterns of associations between anxiety and academic performance vary across four distinct groups of high‐achieving adolescents (selected for high achievement in Sciences, Arts, Literature, or Sports), by examining anxiety–performance links within each group separately.

## 2 Method

### 2.1 Participants

The sample consisted of 800 students aged 12–18 years old (M_age_ = 15.26; SD_age_ = 1.05), with 362 males (M_age_ = 14.93, SD_age_ = 1.03) and 438 females (M_age_ = 15.54, SD_age_ = 0.97) before screening for outliers (see Data Analysis section). Participants were recruited in several educational centres for high performing adolescents in the Russian Federation. Adolescents are selected based on their achievement in Science (e.g., first place in a subject Olympiad; *n* = 366), Arts (e.g., first place in a music competition; *n* = 136), Sports (e.g., a member of an elite sport team; *n* = 132), and Literature (e.g., a poetry prize; *n* = 147) to complete 1‐month educational programmes in these centres. The data was collected during the programme.

### 2.2 Measures

Eight anxiety measures were selected for the present study to capture a wide range of anxiety‐related contexts: 4 general anxiety measures: trait and state anxiety, generalized anxiety disorder, and worry; and 4 measures of specific anxiety types: maths anxiety, social anxiety, spatial anxiety, and anxiety sensitivity. All measures were adapted to Russian using the procedure of translation and back translation following the International Test Commission guidelines for test translations (International Test Commission 2017).

All measures are widely used and have shown high reliability in previous research. Table 1 presents example items, internal consistency statistics for each measure in the present sample as well as the two‐week test–retest reliabilities reported in previous studies.

**TABLE 1:** Description of the measures.

Measure	Type of anxiety	Time frame for items^a^	No of items	Example item	Answer scale	*α*	Two week test—retest reliability (r)
State trait anxiety inventory‐trait—STAIT (Spielberger 1972)	General	Generally	20	*I want to cry*	1 (almost never) 4 (almost always)	0.88	0.88 (Barnes et al. 2002)
State trait anxiety inventory‐state—STAIS (Spielberger 1972)	General	Right now	20	*I am tense*	1 (not at all) 4 (very much so)	0.89	0.70 (Barnes et al. 2002)
Abbreviated maths anxiety scale—AMAS (Hopko et al. 2003)	Math	Generally	9	*Having to use the tables in the back of a maths book*	1 (low anxiety) 5 (high anxiety)	0.82	0.85 (Hopko et al. 2003)
Generalized anxiety disorder questionnaire—GAD (Spitzer et al. 2006)	General	Last 2 weeks	7	*Feeling nervous, anxious or on edge*	0 (not at all) 3 (nearly every day)	0.81	0.83 (Spitzer et al. 2006)
Childhood anxiety sensitivity index—CASI (Silverman et al. 1991)	Bodily symptoms	Generally	18	*I don't want other people to know when I feel afraid*	1 (none) 3 (a lot)	0.94	*0*.76 (Silverman et al. 2003)
Child spatial anxiety questionnaire—SAnx (Ramirez et al. 2012)	Spatial	Generally	8	*How do you feel being asked to say which direction is right or left?*	1 (not nervous) 8 (nervous)	0.82	0.56 (Ramirez et al. 2012)
Appraisal of social concerns questionnaire—ASC (Telch et al. 2004)	Social	Generally	20	*What best describes the degree to which you would be concerned in social situations when: People ignoring you*.	0 (not at all concerned) 100 (extremely concerned)	0.84	0.82 (Telch et al. 2004)
Penn state worry questionnaire—PSWQ (Meyer et al. 1990)	General	Generally	16	*My worries overwhelm me*	1 (absolutely not typical of me) 5 (very typical of me)	0.83	*0*.54 (Hopko et al. 2003)

*Note:* *α*—Cronbach's alphas are specific to the present sample. Two‐week reliability *r* come from different sources. Total scores for the measures were obtained by summing up all items of a respective questionnaire.

^a^ The temporal context of the questionnaire, that is, how the questions were framed, asking, for example, how person generally feels.

In addition, participants reported their teacher‐assigned grades in 13 school subjects: Russian, Algebra, Geometry, English, Literature, Information Technologies (IT), History, Geography, Biology, Sociology, Physics, Chemistry, and Astronomy. These grades are assigned at the end of the school year by teachers and vary from 1 to 5, with grades starting from 3 being “pass” (satisfactory‐good‐excellent). Grades 1 and 2 are usually “fail” grades but are used rarely (see Budakova et al. 2021; Likhanov et al. 2021 for discussion on the lack of sensitivity of this scale).

### 2.3 Procedure

Ethics Committee for Interdisciplinary Investigations at the Tomsk State University (code of ethical approval: 16012018‐5) approved the study. No specific exclusion criteria were applied beyond the selection process for the educational program itself. Participants' assents were obtained on the day of data collection, and their parents' or guardians' written informed consent was obtained before the beginning of an educational programme. The information on the aims and procedures was provided together with admission documents for the programme. No compensation was provided for participation in the study.

Participants completed computerized self‐report questionnaires in a computer room in groups up to 25 people. All questionnaires were administered in the same order. The present paper focuses on eight measures out of a larger battery of tests, which lasted 1.5 h.

### 2.4 Data Analysis

Descriptive statistics, bivariate correlations, and Principal Component Analysis (PCA; Jackson 1991) were calculated using the JASP statistical package (v. 0.17.1). Confirmatory Factor Analysis (CFA; Brown 2015) was run in R‐studio version 3.5.2, using lavaan package (https://lavaan.ugent.be/). Nineteen participants were excluded from the analyses as the data on gender were missing. Univariate outliers were identified by z‐scoring all measures (Field et al. 2012)—any observation larger than an absolute value of 3.29 was excluded from further analysis (5 outliers from GAD, 55 from Russian, and 43 from Algebra grades). Following this, 25 multivariate outliers were identified and excluded using Mahalahobis distance (Field et al. 2012). As participants could skip items, data for some items were missing (less than 5% of cases). No imputation methods were applied. The main analyses were run on scores from 782 participants (420 females).

All variables were normally distributed with absolute skewness values not greater than 2 and with absolute kurtosis values not greater than 7 (Curran et al. 1996). The internal consistency of all questionnaires and inventories was in the acceptable range as per Cronbach's alpha coefficient (Peterson 1994) and are presented in Table 1.

## 3 Results

### 3.1 Descriptive Statistics and Bivariate Correlations

Descriptive statistics for anxiety measures for the full sample are presented in Table 2. Overall, our data showed that the average level of anxiety as measured by all 8 measures was relatively low and consistent with that reported in previous literature in nonclinical adolescent and adult samples. See Table S1 for exact means and references from the respective studies. Descriptive statistics for all study variables (8 anxiety measures and 13 grades divided by gender and area of expertise) are available in Table S2A,B.

**TABLE 2:** Descriptive statistics.

Measure	M	SD	Kurtosis	Skewness	Actual range	Possible range	Mean (SD) from previous studies
STAIT	39.73	9.06	−0.35	0.37	20–65	20–80	25.88 (9.48); (Vera‐Villarroel et al. 2007)
STAIS	35.56	8.29	0.35	0.87	20–56	20–80	22.10 (10.64); (Vera‐Villarroel et al. 2007)
GAD	3.85	3.57	1.16	1.14	0–13	0–21	61% of participants demonstrated a score from 0 to 4; (Victor Mbanuzuru et al. 2023)
AMAS	15.77	5.29	0.09	0.73	9–34	9–45	14.23 (0.92); Marakshina, Pavlova, et al., 2023
ASC	34.67	18.15	0.73	0.03	0–90.05	0–100	52.96 (20.15)^a^; (Schultz et al. 2006)
SAnx	2.37	1.01	−0.66	0.54	1–5.38	1–8	7.13 (2.95)^b^; (Ramirez et al. 2012)
CASI	27.01	5.89	−0.52	0.49	18–44	18–54	27.66 (5.73) (Ginsburg and Drake 2002)
PSWQ	44.72	10.85	−0.47	0.26	20–74	16–80	42.2 (11.5)^c^; (Gillis et al. 1995)

Abbreviations: AMAS, abbreviated maths anxiety scale; ASC, appraisal of social concerns questionnaire; CASI, childhood anxiety sensitivity index; GAD, generalized anxiety disorder questionnaire; M, mean; PSWQ, Penn State worry questionnaire; SAnx, child spatial anxiety questionnaire; SD, standard deviation; STAIS, the state–trait anxiety inventory—state; STAIT, the state–trait anxiety inventory—trait.

^a^ 204 treatment‐seeking individuals who met DSM‐IV criteria for social anxiety disorder.

^b^ The scores for this questionnaire could not be compared directly with this study as a 7‐point response scale was used in the current study compared to Ramirez et al. study who used a 17‐point response scale. Sample was also younger (Mage = 7.05 years).

^c^ Adult sample, with age ranging from 18 to 76.

Bivariate correlations across anxiety measures are presented in Table 3. The correlations ranged from 0.25 to 0.67. Correlations across anxiety measures within expert samples are presented in Figures S1–S4 in SOM and showed comparable results. Table S4 presents bivariate correlations across 8 anxiety measures and 13 school grades.

**TABLE 3:** Bivariate correlations between all anxiety measures.

Measure	1	2	3	4	5	6	7
1. STAIT	—						
2. STAIS	0.67**	—					
3. GAD	0.63**	0.63**	—				
4. AMAS	0.37**	0.29**	0.33**	—			
5. ASC	0.53**	0.42**	0.43**	0.37**	—		
6. SAnx	0.35**	0.30**	0.25**	0.26**	0.31**	—	
7. CASI	0.50**	0.41**	0.44**	0.42**	0.60**	0.26**	—
8. PSWQ	0.67**	0.50**	0.56**	0.47**	0.56**	0.27**	0.57**

*Note:* All correlations rounded to two decimal places; **p < 0.01. Table S2 presents correlations across anxiety measures and 13 school grades.

Abbreviations: AMAS, abbreviated maths anxiety scale; ASC, appraisal of social concerns questionnaire; CASI, childhood anxiety sensitivity index; GAD, generalized anxiety disorder questionnaire; PSWQ, Penn State worry questionnaire; SAnx, child spatial anxiety questionnaire; STAIS, the state–trait anxiety inventory—state; STAIT, the state–trait anxiety inventory—trait.

### 3.2 Factorial Structure

#### 3.2.1 Principal Component Analysis (PCA)

PCA was conducted on 8 anxiety measures. The first principal component had an eigenvalue over Kaiser's criterion of 1 and explained 51.0% of the variance. Table 4 shows the factor loadings after rotation, as well as relatively high uniqueness values for each measure.

**TABLE 4:** Component matrix.

Measure	Factor loadings	Uniqueness
The state trait anxiety inventory—trait	0.83	0.31
The state trait anxiety inventory—state	0.72	0.49
Abbreviated maths anxiety scale	0.60	0.63
Generalized anxiety disorder questionnaire	0.74	0.45
Childhood anxiety sensitivity index	0.72	0.48
Child spatial anxiety questionnaire	0.46	0.79
Appraisal of social concerns questionnaire	0.74	0.45
Penn state worry questionnaire	0.82	0.32

*Note:* Uniqueness reflects the percentage of the variance of each variable that is not explained by the component. The Kaiser‐Meyer‐Olkin (KMO) measure verified the sampling adequacy for the analysis, KMO = 0.89 (this value is considered excellent as per guidelines; (Hutcheson 1999)). Bartlett's test of sphericity, χ2(28) = 2464.06, *p* < 0.001, indicated that correlations between scores were sufficiently large for PCA. Oblique (direct oblimin) rotation was performed, as we expected that potential anxiety factors would correlate.

#### 3.2.2 Confirmatory Factor Analysis (CFA)

CFA was used to test the factor structure observed from PCA against 2 additional models based on the literature. We tested three models: (1) a 1‐factor “Anxiety” model with all anxiety measures loading on one general factor; (2) a 2‐factor “General versus Specific” model with STAIT, STAIS, GAD, and PSWQ loading on a “General anxiety” factor; and spatial, maths, social, and anxiety sensitivity (as presumably tapping into anxiety in specific situations/domains) loading on a “Specific anxiety” factor; and (3) a 2‐factor “STEM versus General anxiety” model with maths and spatial anxiety measures loading on a “STEM anxiety” factor, and all other measures loading on a “General anxiety” factor. The third model was specified based on the finding that maths and spatial anxiety measures had the largest unexplained variance as per uniqueness metrics (see Table 4) and close links between space and maths domains (Hawes and Ansari 2020).

Our data showed that the “General versus Specific” was the best fitting model: it had the lowest AIC and BIC and assumptions met for all metrics, except RMSEA (Table 5). Table S6 presents factor loadings for this model, results from other models available from authors on request.

**TABLE 5:** Fit indices for 3 models.

Metrics	“Anxiety”	“General vs. Specific”	“STEM vs. General anxiety”
AIC	37387.76	37316.25	37384.11
BIC	37499.64	37432.80	37500.66
Chi squared	194.70***	121.19***	189.05***
RMSEA	0.106***	0.083***	0.107***
TLI	0.900	0.938	0.897
CFI	0.928	0.958	0.930

*Note:* ***p < 0.001; Model 2 and 3 showed significant differences in fit indices. CFA assumptions were as follows: RMSEA from 0.06 to 0.08, TLI ≥ 0.95, CFI ≥ 0.95; (Schreiber et al. 2006).

To further investigate anxiety specificity, we explored factor structure in each expertise group separately. Firstly, we conducted 2 (gender) by 4 (expertise) ANOVA for each anxiety measure. Overall, our data showed that expertise groups differ in STAIT and AMAS only, with negligible effect sizes (See Table S3).

The PCA was conducted for each group separately as previous research showed significant differences among these groups on a number of factors, including academic achievement, personality, creativity and spatial ability (Papageorgiou et al. 2020; Repeykova et al. 2023; Tsigeman et al. 2023). We conducted a PCA with oblimin rotation, allowing factors to correlate. While the unifactorial structure replicated in Science and Arts groups, a bifactorial structure emerged in Sports and Literature groups, with result close to “General versus Specific” model (see Table 6).

**TABLE 6:** Factor loadings for 8 anxiety measures within sport and literature groups.

	Sport		Literature	
	Factor 1	Factor 2	Factor 1	Factor 2
	“General”	“Specific”	“General”	“Specific”
STAIT	0.833		0.796	
STAIS	0.761		0.743	
GAD	0.592		0.711	
PSWQ^a^	0.482		0.587	
SAnx	0.382			0.388
ASC^b^	0.316	0.434		0.559
CASI		0.878		0.857
AMAS		0.342		0.363
Explained variance (after rotation)	0.29	0.18	0.28	0.20
Cumulative variance	0.47		0.48	

*Note:* Applied rotation method is oblimin.

Abbreviations: AMAS, abbreviated maths anxiety scale; ASC, appraisal of social concerns questionnaire; CASI, childhood anxiety sensitivity index; GAD, generalized anxiety disorder questionnaire; PSWQ, Penn State worry questionnaire; SAnx, child spatial anxiety questionnaire; STAIS, the state–trait anxiety inventory—state; STAIT, the state–trait anxiety inventory—trait.

^a^ Cross‐loading with larger contribution to Factor 1 in both Sport and Literature samples.

^b^ Cross‐loading with larger contribution to Factor 2 in Sport group.

### 3.3 Links Between Anxiety Measures and School Grades

Table 7 presents results of regression analyses, with 8 anxiety measures, gender, age and expertise domain as predictors and grade as an outcome. This model was repeated for each of the 13 grades. We assessed multicollinearity in the regression models by calculating Variance Inflation Factors (VIFs). All VIF values were below 2.5—under the common threshold of 5–10 (O'brien 2007), indicating that multicollinearity was not a concern for the interpretation of the regression coefficients.

**TABLE 7:** Multiple regression models predicting 13 grades from 8 anxiety measures, Expertise domain, age and gender.

Predictors	Outcomes												
	Algebra	Geometry	Russian	English	Literature	IT	History	Geography	Biology	Sociology	Physics	Chemistry	Astronomy
STAIT	−0.04	−0.02	−0.04	−0.03	−0.07	0.01	0.04	−0.03	−0.10	0.04	−0.04	−0.09	0.03
STAIS	−0.07	−0.09	−0.08	−0.09*	−0.00	−0.07	−0.15***	−0.11*	−0.04	−0.12*	−0.05	−0.05	0.08
AMAS	−0.19***	−0.19***	−0.05	−0.02	−0.04	−0.06	0.02	0.01	−0.01	0.04	−0.03	−0.07	0.11
GAD	0.02	0.02	0.00	−0.07	−0.01	0.00	−0.01	0.02	0.04	−0.03	−0.00	0.01	0.04
ASC	0.06	0.04	−0.02	0.02	0.03	0.02	−0.04	0.02	0.05	−0.01	−0.04	0.04	−0.02
CASI	0.02	0.02	0.04	0.05	−0.00	0.07	0.00	−0.01	−0.02	0.02	−0.01	−0.02	0.03
SAnx	−0.10**	−0.10**	−0.09*	−10**	−0.03	−0.05	−0.06	−0.09*	−0.04	−0.05	−0.06	−0.11**	−0.24*
PSWQ	0.09	0.11*	0.11*	0.03	0.05	−0.05	0.07	0.11*	0.07	−0.00	0.05	0.16**	0.02
Exp arts^a^	−0.30***	−0.31***	−0.10*	−0.05	−0.03	−0.13**	−0.19***	−0.08	−0.18***	−0.18***	−0.19***	−0.37***	−0.11
Exp sports^a^	−0.48	−0.52	−0.38	−0.37	−0.19	−0.25	−0.32	−0.33	−0.36	−0.28	−0.49	−0.61	−0.36
Exp literature^a^	−0.05	−0.02	0.07	0.03	0.11*	−0.02	0.03	−0.02	−0.06	0.01	−0.09	−0.16**	−0.07
Gender (F)^b^	0.05	0.07	0.22***	0.16***	0.22***	0.09**	0.15***	0.10*	0.16***	0.11**	0.06	0.03	0.17*
Age	0.00	−0.05	0.06	0.08*	−0.07	−0.06	0.00	0.06	0.01	0.01	−0.01	−0.00	−0.07
F	16.15***	17.14***	19.09***	13.90***	9.06***	5.64***	10.80***	9.66***	9.17***	7.13***	10.19***	11.40***	2.95***
R^2^	0.22	0.23	0.25	0.20	0.14	0.09	0.16	0.15	0.14	0.12	0.16	0.20	0.23
*N*	741	741	741	741	741	715	740	727	738	723	732	604	147

*Note:* *p < 0.05, **p < 0.01, ***p < 0.001.

Abbreviations: AMAS, abbreviated maths anxiety scale; ASC, appraisal of social concerns questionnaire; CASI, childhood anxiety sensitivity index; GAD, generalized anxiety disorder questionnaire; PSWQ, Penn State worry questionnaire; SAnx, child spatial anxiety questionnaire; STAIS, the state–trait anxiety inventory—state; STAIT, the state–trait anxiety inventory—trait.

^a^ Unstandardized coefficient, with Science track being a baseline, a negative value means that Science track had higher score. All coefficients are significant for Exp Sports at *p* < 0.001.

^b^ Unstandardized coefficient, with males being a baseline, a positive value means that females had higher score.

Our data showed that 4 out of 8 anxiety measures (STAIT, GAD, ASC and CASI) were not associated with any of the grades when other predictors were controlled for. AMAS was the strongest predictor of performance out of all anxiety measures and was linked only with math‐related grades (Algebra and Geometry). Spatial anxiety and STAIS were weakly linked with some grades. PSWQ was the only measure that linked with performance positively. Additionally, as can be seen from Table 7, gender and expertise were the most robust predictors of achievement across domains.

**FIGURE 1:**
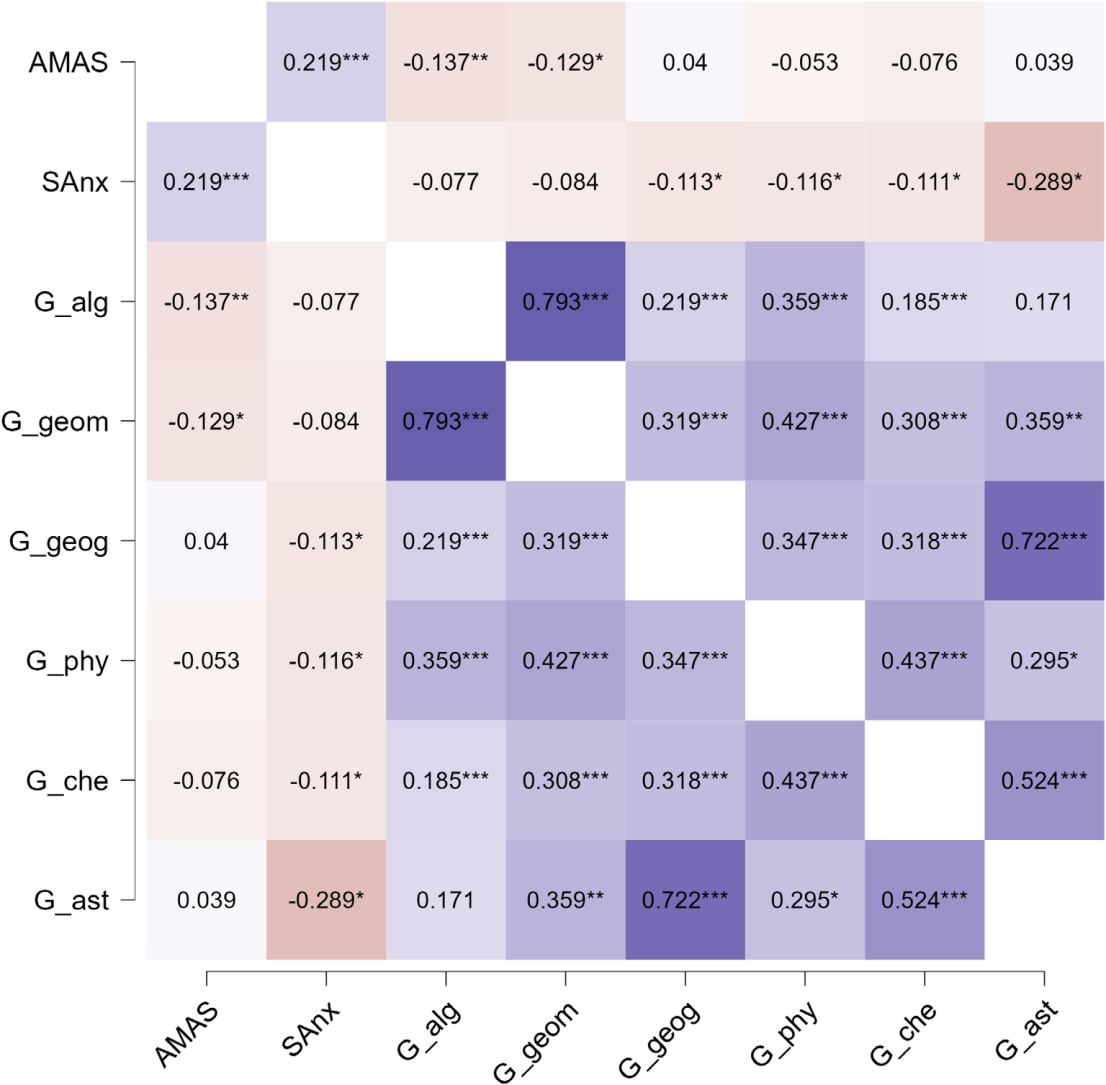
Correlations among STEM year marks and STEM‐related anxieties: Spatial and maths, in science group.

In addition, we explored links among 8 anxiety measures and 13 school grades divided by expertise group (see Figures S1–S4 in SOM for full correlational matrices and Table S5 for ANOVA results on the effects of gender × expertise on achievement). Our data presented a particular opportunity to conduct an analysis of the links between STEM‐related anxiety and achievement in a STEM‐selected sample, which showed that algebra and geometry grades negatively correlated with maths anxiety even in this sample, and spatial anxiety correlated with astronomy, chemistry, physics, and geography in this group (see Figure 1).

## 4 Discussion

Our data showed that 8 anxiety measures correlated positively and at least moderately with each other, and loaded on a single factor. However, this factor explained only 51% of the overall variance, suggesting substantial specificity for each anxiety measure. This specificity was reflected in the correlational patterns among anxiety and school grades. Four of the anxiety measures did not predict grades, and of the remaining four, the strongest negative associations were within domain (e.g., maths anxiety—maths grades). The negative link between maths anxiety and maths performance was found both in the overall sample and in STEM experts, suggesting that anxiety contributes to performance at all levels of achievement. Interestingly, worry correlated positively with performance irrespective of subject domain after controlling for other anxiety types, suggesting that it is distinct from anxiety in its associations with academic performance. The following sections provide a detailed discussion of these results.

### 4.1 Associations Across Anxiety Measures

Although the PCA suggested a single‐factor solution which is consistent with a general anxiety dimension, with the general factor explaining 51% of variance; the CFA analysis favored a two‐factor “General versus Specific Anxiety” structure, with STAIT, STAIS, GAD and PSWQ loading on the “General anxiety” factor, and spatial, maths, social and anxiety sensitivity loading on the “Specific anxiety” factor. This likely reflects differences in the two analytic approaches. PCA is a data‐driven technique that maximizes shared variance and often yields a strong first component when measures are moderately intercorrelated (Jolliffe and Cadima 2016), as was the case in the present sample (*r* = 0.25–0.67). In contrast, CFA tests a priori structural hypotheses and evaluates whether separating constructs improves model fit beyond shared variance (Brown 2015). The better fit of the two‐factor CFA model despite high intercorrelations supports the conclusion that, in addition to a strong general anxiety factor, domain‐specific anxieties (particularly math and spatial anxiety) retain meaningful unique variance.

These results are in line with much previous research (Ferguson et al. 2015; Field et al. 2019; Gibeau et al. 2023; Gillis et al. 1995; Malanchini et al. 2017; Wang et al. 2014), which showed communalities in the used anxiety measures. The results are also consistent with the previous study, which showed a high degree of specificity in generalized, maths, and spatial anxiety and a moderate overlap among the measures, explained by both genetic and environmental factors (Malanchini et al. 2017).

We run additional analyses, splitting the sample into four expert groups. The absolute levels of anxiety were similar for different groups. The pattern of associations among anxiety measures found in the overall sample was replicated in Science and Arts groups. In the Literature and Sports groups, the explained variance was similar to the overall sample, but the factor structure was somewhat different. The Literature group showed that anxiety measures loaded on two factors: presumably a general (STAIT, STAIS, GAD, and PSWQ) versus a specific anxiety factor (ASC, AMAS, SAnx, and CASI); the Sports group also showed a two‐factor but with somewhat different loadings.

The fact that the four general measures loaded on one factor reflects similarity in the items and the latent construct these measures are designed to tap into—anxiety—irrespective of specific situations or domains. The second factor may reflect a more social aspect of anxiety, in particular, in a school context, including embarrassment in front of peers and teachers and negative evaluation by them. Research has suggested that adolescence is a time period when emotional reactivity to the social environment increases and self‐consciousness peaks, with common experiences of embarrassment and shame, including those associated with bodily manifestations of anxiety (Blakemore and Mills 2014; Rankin et al. 2004).

Beyond the social component there are multiple explanations on why specific anxiety measures showed closer links. For example, there is an extensive literature documenting links between spatial and maths anxiety (Ferguson et al. 2015; Lauer et al. 2018; Malanchini et al. 2017), as well as links between spatial and maths domains (studies on mechanisms: Tosto et al. 2014; review on the mechanisms: Hawes and Ansari 2020; meta‐analyses on the link: Atit et al. 2022; Xie et al. 2020; and some individual studies showing that spatial and maths ability could be linked even beyond the general intelligence factor: Likhanov et al. 2024).

Beyond the well‐documented overlap between math and spatial anxiety, our correlation analyses reveal other associations that reflect shared structure. For example, trait anxiety (STAIT) and worry (PSWQ) were highly correlated (*r* = 0.67), consistent with models positioning worry as a core feature of trait anxiety (Meyer et al. 1990; Behar et al. 2009). Similarly, anxiety sensitivity and social anxiety (ASC) showed strong overlap (*r* = 0.60), likely because both capture fear of publicly observable anxiety symptoms (e.g., trembling, blushing) and anticipated negative social evaluation (Silverman et al. 1991; Telch et al. 2004). Worry (PSWQ) also correlated strongly with social concerns and generalized anxiety (*r* = 0.56), supporting its role as a transdiagnostic process that cuts across anxiety domains (McEvoy et al. 2013). These patterns suggest that while a general anxiety factor underpins much of the shared variance, meaningful specificity arises from how different aspects of anxiety link with each other. More fine‐grained (symptom level) research, for example, using network analysis (Birkeland et al. 2017; Fried et al. 2016) is needed to get further insights into intricacies of these links.

### 4.2 Regression Analysis Results

Regression analysis showed that only 4 out of 8 anxiety measures significantly contributed to academic achievement after controlling for other anxiety measures, expertise, and gender. Maths, spatial, and state anxiety contributed negatively, and worry positively to academic achievement in different subjects.

Maths anxiety correlated negatively with math‐related subjects: Algebra and Geometry grades, which is in line with a large body of research (see e.g., a meta‐analysis; Zhang et al. 2019; and reviews: Budakova et al. 2020; Dowker et al. 2016; Li, Chen, et al. 2023; Suárez‐Pellicioni et al. 2016). Maths anxiety also showed weak zero‐order correlations with Russian, English and Chemistry grades, which disappeared when all other anxiety types were controlled. This is in line with a body of research that shows overlap of maths anxiety with both general anxiety and maths performance at phenotypic (Lauer et al. 2018; Sasanguie et al. 2024; Wu et al. 2012) and aetiological level (Malanchini et al. 2020; Wang et al. 2014).

Spatial anxiety correlated negatively with Algebra, Geometry, Geography, Chemistry and Astronomy, which is consistent with a large body of research that showed links between spatial anxiety and performance in spatial domain (Ferguson et al. 2015; Ramirez et al. 2012; Wong 2017). The fact that spatial anxiety was linked with performance in Science is consistent with a wealth of findings on links between spatial ability and performance in STEM, including: maths (e.g., a meta‐analysis, Xie et al. 2020, including geometry); Chemistry (Stieff 2013; Stieff et al. 2014); Geography (Cortes et al. 2022; Dong et al. 2023); and overall science performance (Buckley et al. 2018; Stieff and Uttal 2015; Yan et al. 2023). Spatial anxiety also correlated with two language grades: Russian and English. This is a more general pattern of associations than that observed for maths anxiety, which can potentially be explained by differences in the content of the measures. AMAS explicitly taps into situations related to school maths, for example, “Thinking about a forthcoming maths test”. In contrast, the spatial anxiety measure taps into anxiety experienced in a range of situations where one needs to demonstrate spatial knowledge in front of other people or under time pressure, often expressed verbally. For example, “How do you feel being asked to say which direction is right or left?” or “How do you feel when you have to solve a maze like this in one minute?”. This “fear of verbalizing” may be reflected in these correlations between spatial anxiety and grades in languages.

Only two out of four general anxiety measures were significant independent predictors of academic achievement: STAIS and PSWQ. Interestingly, out of the two scales of the STAI measure it was state (and not trait) scale that correlated with achievement in English, History, Geography and Social studies. Previous research found moderate‐to‐strong correlations between the two scales (e.g., Vigneau and Cormier 2008), suggesting that they tap into substantially overlapping constructs, but focus on stable (trait‐like typical performance) versus “online” (during task performance) anxiety (Spielberger 1970). This division is reflected in the instructions for each scale. However, research into the structure of STAI suggests that the two scales also include items that could be clustered differently. For example, studies demonstrated 3‐factor (state anxiety, usual level of state anxiety, and neuroticism; Barker et al. 1977) and 4‐factor (e.g., State Anxiety Present, State Anxiety Absent, Trait Anxiety Present, Trait Anxiety Absent; Bieling et al. 1998; Vigneau and Cormier 2008) solutions. In our study the correlation between the scales was 0.6, but only STAIS showed relatively robust zero‐order correlations with academic achievement. Examining the items of the two scales suggests that items in the State scale may be more directly relevant to anxiety per se. In contrast, Trait anxiety scale includes items tapping into more general “negative affect” factor (Bados et al. 2010) or even depression (see Elwood et al. 2012 for review). However, it is not clear why it did not contribute to achievement in our study, as previous studies have also found links of such traits with academic achievement (Owens et al. 2012). GAD‐7 also did not contribute to achievement probably because the “more acute” general anxiety it taps into (Alenina et al. 2025) was already accounted for by state anxiety, or because the overall anxiety level (and Generalized Anxiety Disorder symptoms in particular) was quite low in this nonclinical sample.

Recent research suggested that the links between anxiety, depression and academic achievement are complex and are moderated by multiple factors, including attention, working memory and motivation. For example, one study showed that when depression is controlled for, anxiety may be motivating and even leads to higher achievement (McCurdy et al. 2023). Indeed, such moderating processes may help explain the positive associations between academic achievement and the PSWQ scale found in the current study in a regression analysis. Controlling for other anxiety measures, worry was an independent positive predictor of Russian, Geometry, Geography and Chemistry grades. At the same time, zero‐order correlations between worry and grades were mixed—positive or negative—and of a negligible size. Previous research also uncovered this mixed pattern of results, showing zero (Alfonso and Lonigan 2021; Owens et al. 2012), positive (Walkenhorst and Crowe 2009) and negative (Owens et al. 2012) correlations between worry and academic achievement. It is possible that the effect of worry is moderated by other traits. In our study controlling for all other anxieties may have removed variance associated with depression and other negative affect aspects, which may have made “motivational” component of worry more salient.

This explanation is consistent with Yerks‐Dodson's optimal performance theory which suggested that after controlling for the depressive/effective component, worry may act as a form of motivation (Yerkes and Dodson 1908). This effect was previously documented in a study of maths anxiety, which showed that moderate levels of anxiety could be beneficial for maths performance in people with high maths motivation (Wang et al. 2015). Anxiety may act as a positive arousal that helps students with high motivation to focus on their curriculum or studies and thus have better performance. While academic motivation was not measured in the current study, we can hypothesise that it indeed was relatively high, given previous studies in gifted samples that showed higher academic motivation in such samples as compared to their unselected peers (Hornstra et al. 2020; Sternberg and Davidson 2005).

### 4.3 Gender and Expertise Differences in Anxiety and Grades

Females demonstrated slightly higher anxiety for all measures. This is line with a large body of research, including reviews and meta‐analyses (Else‐Quest et al. 2006; Esbjørn et al. 2013; Ferguson et al. 2015; Gibeau et al. 2023; Likhanov et al. 2021; Madjar et al. 2018; Păsărelu et al. 2017; Pestle et al. 2008; Zahn‐Waxler et al. 2008). However, the gender differences were negligible (eta squared ranging from 0.00 for spatial anxiety to 0.04 for worry), possibly reflecting the overall low levels of anxiety in our sample—as in other nonclinical samples.

In our study, females outperformed males in 9 out of 13 school subjects, with negligible effect for 8 subjects (eta squared of 0.01–0.02) and a slightly stronger effect for Astronomy, for which the N was reduced (eta squared of 0.09). No gender differences were found for Algebra, Geometry, Physics, and Chemistry. Overall, the absence of substantial gender differences in school subjects was expected as most participants in the current study were selected for strong academic performance, particularly for STEM and language‐related domains.

Our finding of minimal gender differences in STEM grades among high‐achieving adolescents may appear inconsistent with studies reporting gender gaps at the upper tail of the performance distribution (Hyde et al. 2008; Lindberg et al. 2010). However, this difference might be explained by methodological and contextual factors. First, those studies relied on standardized test scores, which are sensitive to performance variation. In contrast, our performance measures were teacher‐assigned, have restricted range (3–5 in our sample) and may reflect effort, behavior, or compliance in addition to ability, potentially masking true performance differences (Likhanov et al. 2021; Papageorgiou et al. 2020). Second, while our Science‐track participants were selected for high achievement, this selection spanned multiple STEM domains (e.g., biology, informatics, physics), not solely quantitative reasoning, possibly diluting gender effects in specific subject.

Overall, these results suggest that for adolescents selected for high achievement, the reported patterns of associations among anxiety, performance, and gender do not replicate. For example, we did not find females' underperformance in maths and other STEM domains (Yu et al. 2023; Yuan et al. 2019) or support for findings reported in a recent paper on numeracy and neuroticism in females (Lunardon et al. 2022), and we did not find higher anxiety in females, which has been suggested to partially explain their underperformance in these domains (Ferguson et al. 2015). Similarly, we did not find a general female advantage in school grades, which has been attributed to motivational, personality, and verbal performance factors (Gustavsen 2019; Houtte 2004; Likhanov et al. 2021; Lu et al. 2023; Steinmayr and Spinath 2008; Wong et al. 2002).

Finally, we examined differences in anxiety and grades across STEM, Arts, Sports and Literature groups. Negligible differences were found in anxiety measures across the four groups. As expected (Likhanov et al. 2021; Papageorgiou et al. 2020; Repeykova et al. 2023; Tsigeman et al. 2022, 2023), we found differences in grades across the expertise groups (eta squared ranging from 0.02 to 0.13), with the highest results for Science and Literature groups. In line with a recent study in a sample of elite hockey players, the Sports track showed the lowest academic results, which is probably a result of their tight training schedule, fatigue and sport‐related head trauma (see Bartseva et al. 2024 for discussion).

Interestingly, when predicting school grades from 8 anxiety measures, gender and educational track, our data showed female advantage in all subjects, except ones related to mathematics or STEM, which is partly in line with meta‐analytic findings of a female advantage in school grades across subjects (Voyer and Voyer 2014) and with studies showing male overrepresentation in the upper tail of standardized math performance (Contini et al. 2017). These results call for the need to account for anxiety in research of gender differences, as both gender and anxiety contributed to achievement in our study. This is especially important as it was shown that the link between maths anxiety and maths achievement is stronger for girls than for boys (see e.g., a large study of Chinese schoolchildren (*N* = ~28,000; Yu et al. 2023)), suggesting girls may be more sensitive to negative consequences of anxiety.

## 5 Limitations and Future Directions

The current study has some limitations. Firstly, the factor structure identified in our study should be cross‐validated in an independent sample to confirm its stability and generalizability. Secondly, the current study relied on grades assigned by teachers at the end of the school year as a measure of academic achievement. However, these grades have a restricted range (typically 3–5 in high‐achieving samples) and may be susceptible to teacher bias, which limits their sensitivity to detect fine‐grained differences in performance (Budakova et al. 2021; Likhanov et al. 2021; Papageorgiou et al. 2020). This skew could have attenuated the observed anxiety–performance correlations and may explain why some theoretically expected associations (e.g., involving general anxiety measures) did not reach significance. Future studies should complement or replace these grades with standardized exam scores or cognitive performance tasks to improve measurement precision.

Thirdly, further research is needed to evaluate how the observed pattern of results applies to other domains and other types of anxiety, such as performance anxiety in music (Osborne and Kenny 2005), second language (Tsigeman et al. 2026), sport (Smith et al. 2006), test anxiety (Szafranski et al. 2012), as well as aging anxiety (Le Tirant et al. 2026). Fourthly, further research is also needed to investigate whether the observed patterns of associations generalize to other samples of adolescents, for example, not selected for achievement or selected for achievement in other domains. In particular, it is important to explore why STAIT, GAD, ASC and CASI were not significant predictors of any of the school subjects after controlling for other measures of anxiety. This will help with further understanding of the mechanisms of anxiety‐performance associations, such as the role of performance and motivation tilt (i.e., higher motivation in some subjects compared to other ones; Becker et al. 2022). In high‐achieving students selected for high achievement in a specific domain, such motivational asymmetries may moderate the impact of domain‐specific anxiety on performance.

Fifthly, the current study used the total score for each anxiety measure, as is conventional in the field of research into the links between anxiety and performance, especially when multiple anxiety measures are included. However, item‐level approaches may be needed to clarify and explain the observed patterns of associations. For example, there are some items in the spatial anxiety measure that capture spatial anxiety in social situations, which is not the case for other measures of specific anxiety. Similarly, the maths anxiety measure used in this study included items tapping into anxiety related to test situations, as well as to classroom and self‐study learning contexts—which may elicit different anxiety responses in different students. For example, a student may experience anxiety during maths class because they worry about social evaluation and their own reaction to it, because they worry about failing their own expectations and the expectations of significant others, and because they underperform on timed tests.

Sixthly, different anxiety measures vary in extent to which they tap into rumination, shame, guilt, depression and other related constructs (Alenina et al. 2025; Heeren et al. 2018). Further research is needed to establish how the observed specificity is driven by these general constructs versus the domain‐specific context. A fruitful direction for further research might be applying machine learning or Large Language Models approaches to specific items from multiple anxiety measures, searching for clusters of items within/across existing measures.

Lastly, studies that investigate links between different anxiety types and various outcomes in experimental or semi‐experimental designs might shed light on some specific effects (see, e.g., a recent study which compared effects of general vs. maths anxiety on fatigue as reflected in EEG patterns; Zhban et al. 2018 or compared teacher effects on geography versus maths anxiety in schoolchildren; White et al. 2024). Additionally, eye‐tracking methodologies have been used to reveal attentional biases in anxious individuals during academic tasks (e.g., avoidance of math‐related stimuli in high math‐anxiety participants; Li, Quintero, et al. 2023).

## 6 Conclusion

Overall, this study demonstrated that even among high‐achieving adolescents, anxiety remains a meaningful predictor of academic achievement, but the effect was mostly demonstrated for domain‐specific anxiety rather than domain‐general anxiety. While a strong general anxiety factor underlies all measures, maths and spatial anxieties uniquely predicted lower grades in maths and STEM‐related subjects, underscoring the importance of context‐specific assessment and interventions. Surprisingly, worry was positively associated with performance (when general anxiety was controlled for), suggesting it may reflect adaptive cognitive engagement rather than debilitative anxiety in motivated students.

Better understanding of anxiety constructs and their links with performance can help researchers and practitioners with the development of better measures and student assessments. This can also aid efforts towards individualized academic accommodations, such as allowing students extra time during tests and other adjustments to test‐taking conditions; and alternative assessments that do not require speaking in front of the class, in addition to general wellbeing and academic support.

## Conflicts of Interest

The authors declare no conflicts of interest.

## Supporting information


**Data S1:** Supporting Information.

## Data Availability

The data that support the findings of this study are available on request from the corresponding author. The data are not publicly available due to privacy or ethical restrictions.
