# Correction to “Anxiety and Performance in High‐Achieving Adolescents: Associations Among 8 General and Specific Anxiety Measures and 13 School Grades”

**DOI:** 10.1002/pchj.70093

**Published:** 2026-04-05

Likhanov, M., E. Alenina, T. Bloniewski, X. Zhou, and Y. Kovas. 2026. “Anxiety and Performance in High‐Achieving Adolescents: Associations Among 8 General and Specific Anxiety Measures and 13 School Grades.” *PsyCh Journal* 15: e70088. https:/doi.org/10.1002/pchj.70088.

In the originally published version of this article, the author names were incorrectly presented with the surname preceding the given name. As a result, the author names were displayed incorrectly in several locations within the article.

The following sections have been corrected.

**Author byline**

The author byline should read:

Maxim Likhanov^1^ | Evgenia Alenina^2^ | Tomasz Bloniewski^3^ | Xinlin Zhou^4^ | Yulia Kovas^5^

**Correspondence**

The correspondence information should read:

Correspondence: Maxim Likhanov (maksim.likhanov@univ-amu.fr)

**Equal contribution footnote**

The equal contribution statement should read:

“Maxim Likhanov and Evgenia Alenina contributed equally to this study.”

These errors have been corrected in the online version of the article.

We apologize for these errors.

