# Supplementary material for: Anxiety and Performance in High‐Achieving Adolescents: Associations Among 8 General and Specific Anxiety Measures and 13 School Grades
Source: Psych J. 2026 Mar 12;15(2):e70088. doi: 10.1002/pchj.70088 (PMC13140474; doi:10.1002/pchj.70088)
Supplement: Supplementary file 1 — Data S1: Supporting Information. [file PCHJ-15-e70088-s001.docx]

# **Supplementary materials**

# **Table S1. Descriptive statistics with Mean and SD from previous studies.**

| **Measure** | **M** | **SD** | **Two-week test -retest reliability (r)** | **Mean (SD) from previous studies** |
| --- | --- | --- | --- | --- |
| STAIT | 39.73 | 9.06 | .88 (Barnes et al., 2002) | 25.88 (9.48); (Vera-Villarroel et al., 2007) |
| STAIS | 35.56 | 8.29 | .88 (Barnes et al., 2002) | 22.10 (10.64); (Vera-Villarroel et al., 2007) |
| GAD | 3.85 | 3.57 | .83 (Spitzer et al., 2006) | 61% of participants demonstrated a score from 0 to 4; (Victor Mbanuzuru et al., 2023) |
| AMAS | 15.77 | 5.29 | .85 (Hopko et al., 2003) | 14.23 (0.92); (Marakshina, Pavlova, et al., 2023) |
| ASC | 34.67 | 18.15 | .82 (Telch et al., 2004) | 52.96 (20.15)*; (Schultz et al., 2006) |
| SAnx | 2.37 | 1.01 | .56 (Ramirez et al., 2012) | 7.13 (2.95)**; (Ramirez et al., 2012) |
| CASI | 27.01 | 5.89 | *.*76 (Silverman et al., 2003) | 27.66 (5.73) (Ginsburg & Drake, 2002) |
| PSWQ | 44.72 | 10.85 | *.*54 (Hopko et al., 2003) | 42.2 (11.5)***; (Gillis et al., 1995) |

***Note:*** M = mean; SD = standard deviation; * 204 treatment-seeking individuals who met DSM-IV criteria for social anxiety disorder; ** the scores for this questionnaire could not be compared directly with this study as a 7-point response scale was used in the current study compared to Ramirez et al., study who used 17-point response scale. Sample was also younger (Mage = 7.05 years); *** adult sample, with age ranging from 18 to 76; STAIT - The State-Trait Anxiety Inventory – Trait, STAIS - The State-Trait Anxiety Inventory – State, GAD - Generalized Anxiety Disorder questionnaire, AMAS - Abbreviated Maths Anxiety Scale, ASC - Appraisal of Social Concerns questionnaire, SAnx - Child Spatial Anxiety questionnaire, CASI - Childhood Anxiety Sensitivity Index, PSWQ - Penn State Worry Questionnair

# **Table S2 A Descriptive statistics for study variables in males**

|  | | **STAIT** | | | | | | | |
| --- | --- | --- | --- | --- | --- | --- | --- | --- | --- |
|  | | **Science** | | **Art** | | **Sports** | | **Literature** | |
| Valid |  | 208 |  | 29 |  | 109 |  | 16 |  |
| Mean |  | 36.889 |  | 37.793 |  | 33.908 |  | 40.500 |  |
| Std. Deviation |  | 8.359 |  | 9.890 |  | 7.367 |  | 10.583 |  |
| Skewness |  | 0.421 |  | 0.349 |  | 1.052 |  | 0.907 |  |
| Kurtosis |  | -0.268 |  | -0.841 |  | 1.112 |  | -0.141 |  |
| Minimum |  | 20.000 |  | 23.000 |  | 21.000 |  | 27.000 |  |
| Maximum |  | 59.000 |  | 56.000 |  | 56.000 |  | 62.000 |  |
|  | | **STAIS** | | | | | | | |
| Valid |  | 206 |  | 29 |  | 108 |  | 15 |  |
| Mean |  | 32.820 |  | 32.897 |  | 33.296 |  | 35.400 |  |
| Std. Deviation |  | 7.725 |  | 9.991 |  | 7.185 |  | 10.232 |  |
| Skewness |  | 0.766 |  | 0.613 |  | 0.911 |  | 0.815 |  |
| Kurtosis |  | 0.416 |  | -0.925 |  | 0.704 |  | -0.579 |  |
| Minimum |  | 20.000 |  | 20.000 |  | 20.000 |  | 24.000 |  |
| Maximum |  | 56.000 |  | 53.000 |  | 54.000 |  | 55.000 |  |
|  | | **AMAS** | | | | | | | |
| Valid |  | 208 |  | 29 |  | 109 |  | 16 |  |
| Mean |  | 13.125 |  | 16.069 |  | 15.679 |  | 14.313 |  |
| Std. Deviation |  | 4.514 |  | 5.719 |  | 5.256 |  | 4.949 |  |
| Skewness |  | 1.494 |  | 0.845 |  | 0.788 |  | 1.329 |  |
| Kurtosis |  | 2.384 |  | -0.220 |  | 0.174 |  | 1.447 |  |
| Minimum |  | 9.000 |  | 9.000 |  | 9.000 |  | 10.000 |  |
| Maximum |  | 31.000 |  | 30.000 |  | 32.000 |  | 27.000 |  |
|  | | **GAD** | | | | | | | |
| Valid |  | 206 |  | 29 |  | 108 |  | 15 |  |
| Mean |  | 3.073 |  | 3.724 |  | 2.713 |  | 3.067 |  |
| Std. Deviation |  | 2.903 |  | 3.369 |  | 2.419 |  | 2.963 |  |
| Skewness |  | 0.963 |  | 0.642 |  | 0.925 |  | 0.799 |  |
| Kurtosis |  | 0.396 |  | -1.118 |  | 0.512 |  | -0.411 |  |
| Minimum |  | 0.000 |  | 0.000 |  | 0.000 |  | 0.000 |  |
| Maximum |  | 13.000 |  | 10.000 |  | 11.000 |  | 9.000 |  |
|  | | **ASC** | | | | | | | |
| Valid |  | 208 |  | 29 |  | 109 |  | 16 |  |
| Mean |  | 27.992 |  | 30.576 |  | 29.596 |  | 32.709 |  |
| Std. Deviation |  | 17.384 |  | 16.055 |  | 17.873 |  | 16.195 |  |
| Skewness |  | 0.200 |  | -0.258 |  | 0.243 |  | 0.099 |  |
| Kurtosis |  | -1.099 |  | -0.933 |  | -1.047 |  | -0.758 |  |
| Minimum |  | 0.000 |  | 1.500 |  | 0.000 |  | 7.000 |  |
| Maximum |  | 68.750 |  | 56.350 |  | 69.100 |  | 60.800 |  |
|  | | **CASI** | | | | | | | |
| Valid |  | 208 |  | 29 |  | 109 |  | 16 |  |
| Mean |  | 25.413 |  | 26.793 |  | 24.284 |  | 26.063 |  |
| Std. Deviation |  | 5.444 |  | 6.061 |  | 5.055 |  | 7.479 |  |
| Skewness |  | 0.719 |  | 0.723 |  | 0.612 |  | 0.935 |  |
| Kurtosis |  | -0.057 |  | 0.419 |  | -0.516 |  | -0.293 |  |
| Minimum |  | 18.000 |  | 18.000 |  | 18.000 |  | 18.000 |  |
| Maximum |  | 42.000 |  | 43.000 |  | 36.000 |  | 41.000 |  |
|  | | **SAnx** | | | | | | | |
| Valid |  | 208 |  | 29 |  | 109 |  | 16 |  |
| Mean |  | 2.272 |  | 2.306 |  | 2.337 |  | 2.008 |  |
| Std. Deviation |  | 1.045 |  | 1.164 |  | 1.023 |  | 0.950 |  |
| Skewness |  | 0.513 |  | 0.580 |  | 0.708 |  | 1.246 |  |
| Kurtosis |  | -1.013 |  | -0.904 |  | -0.105 |  | 1.074 |  |
| Minimum |  | 1.000 |  | 1.000 |  | 1.000 |  | 1.000 |  |
| Maximum |  | 4.750 |  | 4.750 |  | 5.375 |  | 4.125 |  |
|  | | **PSWQ** | | | | | | | |
| Valid |  | 208 |  | 29 |  | 109 |  | 16 |  |
| Mean |  | 39.356 |  | 42.897 |  | 40.661 |  | 45.188 |  |
| Std. Deviation |  | 9.664 |  | 8.809 |  | 6.876 |  | 11.771 |  |
| Skewness |  | 0.354 |  | 0.722 |  | 0.119 |  | -0.085 |  |
| Kurtosis |  | -0.460 |  | 1.366 |  | -0.424 |  | -0.026 |  |
| Minimum |  | 20.000 |  | 28.000 |  | 24.000 |  | 22.000 |  |
| Maximum |  | 66.000 |  | 69.000 |  | 56.000 |  | 66.000 |  |
|  | | **G_rus** | | | | | | | |
| Valid |  | 208 |  | 29 |  | 109 |  | 16 |  |
| Mean |  | 4.558 |  | 4.552 |  | 4.165 |  | 4.875 |  |
| Std. Deviation |  | 0.498 |  | 0.506 |  | 0.373 |  | 0.342 |  |
| Skewness |  | -0.234 |  | -0.220 |  | 1.829 |  | -2.509 |  |
| Kurtosis |  | -1.964 |  | -2.102 |  | 1.370 |  | 4.898 |  |
| Minimum |  | 4.000 |  | 4.000 |  | 4.000 |  | 4.000 |  |
| Maximum |  | 5.000 |  | 5.000 |  | 5.000 |  | 5.000 |  |
|  | | **G_alg** | | | | | | | |
| Valid |  | 208 |  | 29 |  | 109 |  | 16 |  |
| Mean |  | 4.788 |  | 4.448 |  | 4.257 |  | 4.813 |  |
| Std. Deviation |  | 0.409 |  | 0.506 |  | 0.439 |  | 0.403 |  |
| Skewness |  | -1.423 |  | 0.220 |  | 1.128 |  | -1.772 |  |
| Kurtosis |  | 0.025 |  | -2.102 |  | -0.740 |  | 1.285 |  |
| Minimum |  | 4.000 |  | 4.000 |  | 4.000 |  | 4.000 |  |
| Maximum |  | 5.000 |  | 5.000 |  | 5.000 |  | 5.000 |  |
|  | | **G_geom** | | | | | | | |
| Valid |  | 208 |  | 29 |  | 109 |  | 16 |  |
| Mean |  | 4.769 |  | 4.483 |  | 4.220 |  | 4.750 |  |
| Std. Deviation |  | 0.422 |  | 0.574 |  | 0.516 |  | 0.447 |  |
| Skewness |  | -1.287 |  | -0.535 |  | 0.266 |  | -1.278 |  |
| Kurtosis |  | -0.346 |  | -0.663 |  | -0.041 |  | -0.440 |  |
| Minimum |  | 4.000 |  | 3.000 |  | 3.000 |  | 4.000 |  |
| Maximum |  | 5.000 |  | 5.000 |  | 5.000 |  | 5.000 |  |
|  | | **G_eng** | | | | | | | |
| Valid |  | 208 |  | 29 |  | 109 |  | 16 |  |
| Mean |  | 4.707 |  | 4.621 |  | 4.266 |  | 4.750 |  |
| Std. Deviation |  | 0.467 |  | 0.561 |  | 0.538 |  | 0.447 |  |
| Skewness |  | -1.058 |  | -1.164 |  | 0.120 |  | -1.278 |  |
| Kurtosis |  | -0.500 |  | 0.492 |  | -0.395 |  | -0.440 |  |
| Minimum |  | 3.000 |  | 3.000 |  | 3.000 |  | 4.000 |  |
| Maximum |  | 5.000 |  | 5.000 |  | 5.000 |  | 5.000 |  |
|  | | **G_lit** | | | | | | | |
| Valid |  | 208 |  | 29 |  | 109 |  | 16 |  |
| Mean |  | 4.688 |  | 4.655 |  | 4.514 |  | 5.000 |  |
| Std. Deviation |  | 0.475 |  | 0.553 |  | 0.555 |  | 0.000 |  |
| Skewness |  | -0.951 |  | -1.355 |  | -0.553 |  | NaN |  |
| Kurtosis |  | -0.741 |  | 1.044 |  | -0.777 |  | NaN |  |
| Minimum |  | 3.000 |  | 3.000 |  | 3.000 |  | 5.000 |  |
| Maximum |  | 5.000 |  | 5.000 |  | 5.000 |  | 5.000 |  |
|  | | **G_inf** | | | | | | | |
| Valid |  | 207 |  | 23 |  | 108 |  | 16 |  |
| Mean |  | 4.894 |  | 4.696 |  | 4.593 |  | 4.875 |  |
| Std. Deviation |  | 0.309 |  | 0.470 |  | 0.512 |  | 0.342 |  |
| Skewness |  | -2.574 |  | -0.911 |  | -0.594 |  | -2.509 |  |
| Kurtosis |  | 4.669 |  | -1.291 |  | -1.161 |  | 4.898 |  |
| Minimum |  | 4.000 |  | 4.000 |  | 3.000 |  | 4.000 |  |
| Maximum |  | 5.000 |  | 5.000 |  | 5.000 |  | 5.000 |  |
|  | | **G_hist** | | | | | | | |
| Valid |  | 208 |  | 29 |  | 109 |  | 16 |  |
| Mean |  | 4.726 |  | 4.552 |  | 4.385 |  | 4.875 |  |
| Std. Deviation |  | 0.447 |  | 0.686 |  | 0.525 |  | 0.342 |  |
| Skewness |  | -1.021 |  | -1.269 |  | 0.084 |  | -2.509 |  |
| Kurtosis |  | -0.968 |  | 0.411 |  | -1.154 |  | 4.898 |  |
| Minimum |  | 4.000 |  | 3.000 |  | 3.000 |  | 4.000 |  |
| Maximum |  | 5.000 |  | 5.000 |  | 5.000 |  | 5.000 |  |
|  | | **G_geog** | | | | | | | |
| Valid |  | 202 |  | 29 |  | 109 |  | 16 |  |
| Mean |  | 4.752 |  | 4.759 |  | 4.385 |  | 4.813 |  |
| Std. Deviation |  | 0.466 |  | 0.435 |  | 0.592 |  | 0.403 |  |
| Skewness |  | -1.617 |  | -1.276 |  | -0.362 |  | -1.772 |  |
| Kurtosis |  | 1.632 |  | -0.406 |  | -0.677 |  | 1.285 |  |
| Minimum |  | 3.000 |  | 4.000 |  | 3.000 |  | 4.000 |  |
| Maximum |  | 5.000 |  | 5.000 |  | 5.000 |  | 5.000 |  |
|  | | **G_bio** | | | | | | | |
| Valid |  | 208 |  | 29 |  | 109 |  | 16 |  |
| Mean |  | 4.774 |  | 4.586 |  | 4.404 |  | 4.813 |  |
| Std. Deviation |  | 0.452 |  | 0.501 |  | 0.546 |  | 0.403 |  |
| Skewness |  | -1.784 |  | -0.369 |  | -0.128 |  | -1.772 |  |
| Kurtosis |  | 2.278 |  | -2.007 |  | -0.981 |  | 1.285 |  |
| Minimum |  | 3.000 |  | 4.000 |  | 3.000 |  | 4.000 |  |
| Maximum |  | 5.000 |  | 5.000 |  | 5.000 |  | 5.000 |  |
|  | | **G_soc** | | | | | | | |
| Valid |  | 204 |  | 26 |  | 109 |  | 16 |  |
| Mean |  | 4.765 |  | 4.731 |  | 4.505 |  | 4.875 |  |
| Std. Deviation |  | 0.425 |  | 0.533 |  | 0.538 |  | 0.342 |  |
| Skewness |  | -1.257 |  | -1.925 |  | -0.382 |  | -2.509 |  |
| Kurtosis |  | -0.423 |  | 3.200 |  | -1.106 |  | 4.898 |  |
| Minimum |  | 4.000 |  | 3.000 |  | 3.000 |  | 4.000 |  |
| Maximum |  | 5.000 |  | 5.000 |  | 5.000 |  | 5.000 |  |
|  | | **G_phy** | | | | | | | |
| Valid |  | 208 |  | 28 |  | 107 |  | 16 |  |
| Mean |  | 4.788 |  | 4.571 |  | 4.318 |  | 4.750 |  |
| Std. Deviation |  | 0.432 |  | 0.504 |  | 0.525 |  | 0.447 |  |
| Skewness |  | -1.779 |  | -0.305 |  | 0.182 |  | -1.278 |  |
| Kurtosis |  | 2.116 |  | -2.060 |  | -0.752 |  | -0.440 |  |
| Minimum |  | 3.000 |  | 4.000 |  | 3.000 |  | 4.000 |  |
| Maximum |  | 5.000 |  | 5.000 |  | 5.000 |  | 5.000 |  |
|  | | **G_che** | | | | | | | |
| Valid |  | 186 |  | 19 |  | 31 |  | 15 |  |
| Mean |  | 4.866 |  | 4.684 |  | 4.290 |  | 4.533 |  |
| Std. Deviation |  | 0.372 |  | 0.478 |  | 0.643 |  | 0.516 |  |
| Skewness |  | -2.776 |  | -0.862 |  | -0.342 |  | -0.149 |  |
| Kurtosis |  | 7.436 |  | -1.419 |  | -0.585 |  | -2.308 |  |
| Minimum |  | 3.000 |  | 4.000 |  | 3.000 |  | 4.000 |  |
| Maximum |  | 5.000 |  | 5.000 |  | 5.000 |  | 5.000 |  |
|  | | **G_ast** | | | | | | | |
| Valid |  | 43 |  | 5 |  | 9 |  | 3 |  |
| Mean |  | 4.837 |  | 4.600 |  | 4.444 |  | 4.667 |  |
| Std. Deviation |  | 0.374 |  | 0.548 |  | 0.527 |  | 0.577 |  |
| Skewness |  | -1.894 |  | -0.609 |  | 0.271 |  | -1.732 |  |
| Kurtosis |  | 1.661 |  | -3.333 |  | -2.571 |  | NaN |  |
| Minimum |  | 4.000 |  | 4.000 |  | 4.000 |  | 4.000 |  |
| Maximum |  | 5.000 |  | 5.000 |  | 5.000 |  | 5.000 |  |
|  | | **Age** | | | | | | | |
| Valid |  | 208 |  | 29 |  | 109 |  | 16 |  |
| Mean |  | 15.370 |  | 14.828 |  | 14.046 |  | 15.375 |  |
| Std. Deviation |  | 0.954 |  | 1.002 |  | 0.417 |  | 1.147 |  |
| Skewness |  | -0.027 |  | 0.826 |  | 5.000 |  | 0.047 |  |
| Kurtosis |  | -0.997 |  | -0.593 |  | 31.819 |  | -1.432 |  |
| Minimum |  | 14.000 |  | 14.000 |  | 13.000 |  | 14.000 |  |
| Maximum |  | 17.000 |  | 17.000 |  | 17.000 |  | 17.000 |  |

*Note:* *STAIT – State Trait Anxiety Inventory – Trait subscale; State Trait Anxiety Inventory – State subscale; AMAS – Abbreviated Maths Anxiety Scale; GAD - Generalized Anxiety Disorder Questionnaire; ASC – Appraisal of Social Concerns; CASI – Childhood Anxiety Scale; SAnx – Spatial Anxiety; PSWQ – Penn State Worry Questionnaire; G_rus – Russian Language Grade; G_alg – Algebra Grade; G_gem – Geometry Grade; G-eng – English language Grade; G_lit – Literature Grade; G_inf – Informatics Grade; G_hist – History Grade; G_geog – Geography Grade; G_bio – Biology Grade; G-soc – Sociology Grade; G_phy – Physics Grade; G_che – Chemistry Grade; G_ast – Astronomy Grade;*

# **Table S2 B Descriptive statistics for study variables in females**

|  | | **STAIT** | | | | | | | |
| --- | --- | --- | --- | --- | --- | --- | --- | --- | --- |
|  | | **Science** | | **Art** | | **Sports** | | **Literature** | |
| Valid |  | 155 |  | 107 |  | 23 |  | 131 |  |
| Mean |  | 43.594 |  | 44.075 |  | 39.522 |  | 41.328 |  |
| Std. Deviation |  | 8.367 |  | 8.427 |  | 8.190 |  | 8.381 |  |
| Skewness |  | 0.197 |  | 0.133 |  | 1.077 |  | -0.040 |  |
| Kurtosis |  | -0.630 |  | -0.567 |  | 0.623 |  | -0.178 |  |
| Minimum |  | 28.000 |  | 27.000 |  | 30.000 |  | 21.000 |  |
| Maximum |  | 65.000 |  | 63.000 |  | 61.000 |  | 61.000 |  |
|  | | **STAIS** | | | | | | | |
| Valid |  | 152 |  | 100 |  | 23 |  | 123 |  |
| Mean |  | 36.664 |  | 36.280 |  | 33.826 |  | 34.992 |  |
| Std. Deviation |  | 8.909 |  | 8.245 |  | 7.620 |  | 8.080 |  |
| Skewness |  | 0.363 |  | 0.340 |  | 0.407 |  | 0.653 |  |
| Kurtosis |  | -0.791 |  | -0.772 |  | -0.842 |  | -0.037 |  |
| Minimum |  | 21.000 |  | 21.000 |  | 23.000 |  | 21.000 |  |
| Maximum |  | 55.000 |  | 53.000 |  | 48.000 |  | 56.000 |  |
|  | | **AMAS** | | | | | | | |
| Valid |  | 158 |  | 107 |  | 23 |  | 132 |  |
| Mean |  | 16.266 |  | 18.084 |  | 15.565 |  | 17.674 |  |
| Std. Deviation |  | 4.938 |  | 5.522 |  | 3.259 |  | 5.167 |  |
| Skewness |  | 0.849 |  | 0.369 |  | 0.686 |  | 0.244 |  |
| Kurtosis |  | 0.696 |  | -0.673 |  | 1.187 |  | -0.809 |  |
| Minimum |  | 9.000 |  | 9.000 |  | 9.000 |  | 9.000 |  |
| Maximum |  | 34.000 |  | 31.000 |  | 23.000 |  | 30.000 |  |
|  | | **GAD** | | | | | | | |
| Valid |  | 151 |  | 104 |  | 23 |  | 126 |  |
| Mean |  | 4.391 |  | 4.760 |  | 3.522 |  | 4.921 |  |
| Std. Deviation |  | 3.169 |  | 3.331 |  | 2.391 |  | 3.191 |  |
| Skewness |  | 0.622 |  | 0.815 |  | 0.714 |  | 0.687 |  |
| Kurtosis |  | -0.195 |  | 0.167 |  | 0.091 |  | -0.078 |  |
| Minimum |  | 0.000 |  | 0.000 |  | 0.000 |  | 0.000 |  |
| Maximum |  | 13.000 |  | 13.000 |  | 9.000 |  | 13.000 |  |
|  | | **ASC** | | | | | | | |
| Valid |  | 158 |  | 107 |  | 23 |  | 132 |  |
| Mean |  | 40.648 |  | 39.550 |  | 33.246 |  | 39.636 |  |
| Std. Deviation |  | 16.917 |  | 18.639 |  | 16.928 |  | 16.809 |  |
| Skewness |  | -0.307 |  | 0.094 |  | 0.094 |  | -0.036 |  |
| Kurtosis |  | -0.434 |  | -0.269 |  | -1.018 |  | -0.108 |  |
| Minimum |  | 1.750 |  | 0.350 |  | 3.400 |  | 3.350 |  |
| Maximum |  | 75.750 |  | 86.700 |  | 61.650 |  | 90.050 |  |
|  | | **CASI** | | | | | | | |
| Valid |  | 158 |  | 107 |  | 23 |  | 132 |  |
| Mean |  | 29.133 |  | 29.112 |  | 27.130 |  | 27.644 |  |
| Std. Deviation |  | 5.763 |  | 6.334 |  | 5.093 |  | 5.292 |  |
| Skewness |  | 0.131 |  | 0.349 |  | 1.005 |  | 0.435 |  |
| Kurtosis |  | -0.855 |  | -0.789 |  | 1.479 |  | -0.546 |  |
| Minimum |  | 18.000 |  | 18.000 |  | 19.000 |  | 18.000 |  |
| Maximum |  | 42.000 |  | 44.000 |  | 41.000 |  | 42.000 |  |
|  | | **SAnx** | | | | | | | |
| Valid |  | 158 |  | 107 |  | 23 |  | 132 |  |
| Mean |  | 2.536 |  | 2.430 |  | 2.288 |  | 2.376 |  |
| Std. Deviation |  | 1.018 |  | 1.024 |  | 0.834 |  | 0.891 |  |
| Skewness |  | 0.513 |  | 0.624 |  | 0.120 |  | 0.447 |  |
| Kurtosis |  | -0.677 |  | -0.643 |  | -0.927 |  | -0.443 |  |
| Minimum |  | 1.000 |  | 1.000 |  | 1.000 |  | 1.000 |  |
| Maximum |  | 5.000 |  | 4.875 |  | 4.000 |  | 5.000 |  |
|  | | **PSWQ** | | | | | | | |
| Valid |  | 158 |  | 107 |  | 23 |  | 132 |  |
| Mean |  | 49.538 |  | 48.299 |  | 44.826 |  | 48.197 |  |
| Std. Deviation |  | 10.953 |  | 10.651 |  | 8.467 |  | 11.128 |  |
| Skewness |  | -0.100 |  | 0.171 |  | 0.563 |  | -0.016 |  |
| Kurtosis |  | -0.745 |  | -0.534 |  | 1.395 |  | -0.524 |  |
| Minimum |  | 24.000 |  | 23.000 |  | 28.000 |  | 23.000 |  |
| Maximum |  | 74.000 |  | 74.000 |  | 68.000 |  | 73.000 |  |
|  | | **G_rus** | | | | | | | |
| Valid |  | 158 |  | 107 |  | 23 |  | 132 |  |
| Mean |  | 4.848 |  | 4.682 |  | 4.391 |  | 4.886 |  |
| Std. Deviation |  | 0.360 |  | 0.468 |  | 0.499 |  | 0.319 |  |
| Skewness |  | -1.958 |  | -0.794 |  | 0.477 |  | -2.463 |  |
| Kurtosis |  | 1.858 |  | -1.396 |  | -1.951 |  | 4.128 |  |
| Minimum |  | 4.000 |  | 4.000 |  | 4.000 |  | 4.000 |  |
| Maximum |  | 5.000 |  | 5.000 |  | 5.000 |  | 5.000 |  |
|  | | **G_alg** | | | | | | | |
| Valid |  | 158 |  | 107 |  | 23 |  | 132 |  |
| Mean |  | 4.797 |  | 4.495 |  | 4.391 |  | 4.750 |  |
| Std. Deviation |  | 0.403 |  | 0.502 |  | 0.499 |  | 0.435 |  |
| Skewness |  | -1.495 |  | 0.019 |  | 0.477 |  | -1.168 |  |
| Kurtosis |  | 0.237 |  | -2.038 |  | -1.951 |  | -0.646 |  |
| Minimum |  | 4.000 |  | 4.000 |  | 4.000 |  | 4.000 |  |
| Maximum |  | 5.000 |  | 5.000 |  | 5.000 |  | 5.000 |  |
|  | | **G_geom** | | | | | | | |
| Valid |  | 158 |  | 107 |  | 23 |  | 132 |  |
| Mean |  | 4.829 |  | 4.495 |  | 4.391 |  | 4.780 |  |
| Std. Deviation |  | 0.378 |  | 0.521 |  | 0.499 |  | 0.416 |  |
| Skewness |  | -1.766 |  | -0.185 |  | 0.477 |  | -1.370 |  |
| Kurtosis |  | 1.131 |  | -1.508 |  | -1.951 |  | -0.126 |  |
| Minimum |  | 4.000 |  | 3.000 |  | 4.000 |  | 4.000 |  |
| Maximum |  | 5.000 |  | 5.000 |  | 5.000 |  | 5.000 |  |
|  | | **G_eng** | | | | | | | |
| Valid |  | 158 |  | 107 |  | 23 |  | 132 |  |
| Mean |  | 4.816 |  | 4.776 |  | 4.478 |  | 4.902 |  |
| Std. Deviation |  | 0.404 |  | 0.441 |  | 0.593 |  | 0.324 |  |
| Skewness |  | -1.939 |  | -1.672 |  | -0.625 |  | -3.389 |  |
| Kurtosis |  | 2.640 |  | 1.695 |  | -0.470 |  | 11.757 |  |
| Minimum |  | 3.000 |  | 3.000 |  | 3.000 |  | 3.000 |  |
| Maximum |  | 5.000 |  | 5.000 |  | 5.000 |  | 5.000 |  |
|  | | **G_lit** | | | | | | | |
| Valid |  | 158 |  | 107 |  | 23 |  | 132 |  |
| Mean |  | 4.899 |  | 4.888 |  | 4.826 |  | 4.985 |  |
| Std. Deviation |  | 0.303 |  | 0.317 |  | 0.388 |  | 0.123 |  |
| Skewness |  | -2.669 |  | -2.493 |  | -1.843 |  | -8.030 |  |
| Kurtosis |  | 5.188 |  | 4.297 |  | 1.522 |  | 63.438 |  |
| Minimum |  | 4.000 |  | 4.000 |  | 4.000 |  | 4.000 |  |
| Maximum |  | 5.000 |  | 5.000 |  | 5.000 |  | 5.000 |  |
|  | | **G_inf** | | | | | | | |
| Valid |  | 155 |  | 99 |  | 23 |  | 125 |  |
| Mean |  | 4.910 |  | 4.818 |  | 4.913 |  | 4.912 |  |
| Std. Deviation |  | 0.309 |  | 0.388 |  | 0.288 |  | 0.284 |  |
| Skewness |  | -3.532 |  | -1.675 |  | -3.140 |  | -2.944 |  |
| Kurtosis |  | 12.815 |  | 0.823 |  | 8.605 |  | 6.776 |  |
| Minimum |  | 3.000 |  | 4.000 |  | 4.000 |  | 4.000 |  |
| Maximum |  | 5.000 |  | 5.000 |  | 5.000 |  | 5.000 |  |
|  | | **G_hist** | | | | | | | |
| Valid |  | 158 |  | 107 |  | 23 |  | 131 |  |
| Mean |  | 4.880 |  | 4.710 |  | 4.609 |  | 4.916 |  |
| Std. Deviation |  | 0.326 |  | 0.456 |  | 0.499 |  | 0.278 |  |
| Skewness |  | -2.357 |  | -0.940 |  | -0.477 |  | -3.035 |  |
| Kurtosis |  | 3.603 |  | -1.137 |  | -1.951 |  | 7.323 |  |
| Minimum |  | 4.000 |  | 4.000 |  | 4.000 |  | 4.000 |  |
| Maximum |  | 5.000 |  | 5.000 |  | 5.000 |  | 5.000 |  |
|  | | **G_geog** | | | | | | | |
| Valid |  | 156 |  | 107 |  | 23 |  | 126 |  |
| Mean |  | 4.891 |  | 4.785 |  | 4.565 |  | 4.889 |  |
| Std. Deviation |  | 0.313 |  | 0.413 |  | 0.507 |  | 0.316 |  |
| Skewness |  | -2.534 |  | -1.408 |  | -0.282 |  | -2.505 |  |
| Kurtosis |  | 4.479 |  | -0.019 |  | -2.113 |  | 4.343 |  |
| Minimum |  | 4.000 |  | 4.000 |  | 4.000 |  | 4.000 |  |
| Maximum |  | 5.000 |  | 5.000 |  | 5.000 |  | 5.000 |  |
|  | | **G_bio** | | | | | | | |
| Valid |  | 158 |  | 107 |  | 23 |  | 129 |  |
| Mean |  | 4.918 |  | 4.738 |  | 4.652 |  | 4.876 |  |
| Std. Deviation |  | 0.276 |  | 0.462 |  | 0.487 |  | 0.331 |  |
| Skewness |  | -3.070 |  | -1.388 |  | -0.684 |  | -2.308 |  |
| Kurtosis |  | 7.517 |  | 0.673 |  | -1.687 |  | 3.380 |  |
| Minimum |  | 4.000 |  | 3.000 |  | 4.000 |  | 4.000 |  |
| Maximum |  | 5.000 |  | 5.000 |  | 5.000 |  | 5.000 |  |
|  | | **G_soc** | | | | | | | |
| Valid |  | 156 |  | 100 |  | 23 |  | 130 |  |
| Mean |  | 4.910 |  | 4.690 |  | 4.609 |  | 4.908 |  |
| Std. Deviation |  | 0.287 |  | 0.465 |  | 0.499 |  | 0.291 |  |
| Skewness |  | -2.899 |  | -0.834 |  | -0.477 |  | -2.850 |  |
| Kurtosis |  | 6.486 |  | -1.331 |  | -1.951 |  | 6.217 |  |
| Minimum |  | 4.000 |  | 4.000 |  | 4.000 |  | 4.000 |  |
| Maximum |  | 5.000 |  | 5.000 |  | 5.000 |  | 5.000 |  |
|  | | **G_phy** | | | | | | | |
| Valid |  | 158 |  | 106 |  | 23 |  | 127 |  |
| Mean |  | 4.842 |  | 4.660 |  | 4.348 |  | 4.756 |  |
| Std. Deviation |  | 0.366 |  | 0.476 |  | 0.573 |  | 0.431 |  |
| Skewness |  | -1.891 |  | -0.687 |  | -0.132 |  | -1.206 |  |
| Kurtosis |  | 1.596 |  | -1.558 |  | -0.616 |  | -0.555 |  |
| Minimum |  | 4.000 |  | 4.000 |  | 3.000 |  | 4.000 |  |
| Maximum |  | 5.000 |  | 5.000 |  | 5.000 |  | 5.000 |  |
|  | | **G_che** | | | | | | | |
| Valid |  | 153 |  | 94 |  | 19 |  | 124 |  |
| Mean |  | 4.908 |  | 4.511 |  | 4.263 |  | 4.782 |  |
| Std. Deviation |  | 0.311 |  | 0.544 |  | 0.562 |  | 0.414 |  |
| Skewness |  | -3.505 |  | -0.453 |  | 0.058 |  | -1.385 |  |
| Kurtosis |  | 12.596 |  | -0.981 |  | -0.171 |  | -0.084 |  |
| Minimum |  | 3.000 |  | 3.000 |  | 3.000 |  | 4.000 |  |
| Maximum |  | 5.000 |  | 5.000 |  | 5.000 |  | 5.000 |  |
|  | | **G_ast** | | | | | | | |
| Valid |  | 28 |  | 18 |  | 1 |  | 40 |  |
| Mean |  | 4.964 |  | 4.944 |  | 5.000 |  | 4.950 |  |
| Std. Deviation |  | 0.189 |  | 0.236 |  | NaN |  | 0.221 |  |
| Skewness |  | -5.292 |  | -4.243 |  | NaN | ᵃ | -4.292 |  |
| Kurtosis |  | 28.000 |  | 18.000 |  | NaN | ᵃ | 17.285 |  |
| Minimum |  | 4.000 |  | 4.000 |  | 5.000 |  | 4.000 |  |
| Maximum |  | 5.000 |  | 5.000 |  | 5.000 |  | 5.000 |  |
|  | | **Age** | | | | | | | |
| Valid |  | 158 |  | 107 |  | 23 |  | 132 |  |
| Mean |  | 15.437 |  | 15.271 |  | 14.913 |  | 15.992 |  |
| Std. Deviation |  | 0.940 |  | 0.967 |  | 0.848 |  | 0.887 |  |
| Skewness |  | 0.278 |  | -0.316 |  | 0.664 |  | -0.385 |  |
| Kurtosis |  | -0.405 |  | -0.086 |  | 0.019 |  | -0.821 |  |
| Minimum |  | 14.000 |  | 12.000 |  | 14.000 |  | 14.000 |  |
| Maximum |  | 18.000 |  | 17.000 |  | 17.000 |  | 17.000 |  |

Note: *STAIT – State Trait Anxiety Inventory – Trait subscale; State Trait Anxiety Inventory – State subscale; AMAS – Abbreviated Maths Anxiety Scale; GAD - Generalized Anxiety Disorder Questionnaire; ASC – Appraisal of Social Concerns; CASI – Childhood Anxiety Scale; SAnx – Spatial Anxiety; PSWQ – Penn State Worry Questionnaire; G_rus – Russian Language Grade; G_alg – Algebra Grade; G_gem – Geometry Grade; G-eng – English language Grade; G_lit – Literature Grade; G_inf – Informatics Grade; G_hist – History Grade; G_geog – Geography Grade; G_bio – Biology Grade; G-soc – Sociology Grade; G_phy – Physics Grade; G_che – Chemistry Grade; G_ast – Astronomy Grade;*

*Table S2.*

# **Table S3. Gender and expertise differences in 8 anxiety measures**

|  | Effects | | | | | |
| --- | --- | --- | --- | --- | --- | --- |
|  | Gender | | Expertise | | interaction | |
| Anxiety | eta^2 | sig | eta^2 | sig | eta^2 | sig |
| STAIT | .04 | <.001 | .02 | <.01 | .01 | ns |
| STAIS | .01 | <.05 | .00 | ns | .01 | ns |
| AMAS | .02 | <.001 | .02 | <.001 | .01 | ns |
| GAD | .02 | <.001 | .01 | ns | .00 | ns |
| ASC | .02 | <.001 | .00 | ns | .01 | ns |
| CASI | .02 | <.001 | .01 | ns | .00 | ns |
| SAnx | .00 | ns | .00 | ns | .00 | ns |
| PSWQ | .04 | <.001 | .01 | ns | .01 | <.01 |

# **Table S4.** **Correlations across all study variables, controlling for age, gender and expertise**

|  | 1 | 2 | 3 | 4 | 5 | 6 | 7 | 8 | 9 | 10 | 11 | 12 | 13 | 14 | 15 | 16 | 17 | 18 | 19 | 20 |
| --- | --- | --- | --- | --- | --- | --- | --- | --- | --- | --- | --- | --- | --- | --- | --- | --- | --- | --- | --- | --- |
| 1. STAIT | — |  |  |  |  |  |  |  |  |  |  |  |  |  |  |  |  |  |  |  |
| 2. STAIS | .61*** | — |  |  |  |  |  |  |  |  |  |  |  |  |  |  |  |  |  |  |
| 3. AMAS | .33*** | .27*** | — |  |  |  |  |  |  |  |  |  |  |  |  |  |  |  |  |  |
| 4. GAD | .57*** | .56*** | .27*** | — |  |  |  |  |  |  |  |  |  |  |  |  |  |  |  |  |
| 5. ASC | .47*** | .36*** | .32*** | .37*** | — |  |  |  |  |  |  |  |  |  |  |  |  |  |  |  |
| 6. CASI | .41*** | .34*** | .39*** | .36*** | .54*** | — |  |  |  |  |  |  |  |  |  |  |  |  |  |  |
| 7. SAnx | .33*** | .25*** | .26*** | .21*** | .27*** | .23*** | — |  |  |  |  |  |  |  |  |  |  |  |  |  |
| 8. PSWQ | .62*** | .45*** | .40*** | .50*** | .51*** | .51*** | .25*** | — |  |  |  |  |  |  |  |  |  |  |  |  |
| 9. G_rus | -.04 | -.09** | -.07* | -.01 | -.02 | -.00 | -.11*** | .02 | — |  |  |  |  |  |  |  |  |  |  |  |
| 10. G_alg | -.05 | -.09* | -.19*** | -.01 | -.00 | -.03 | -.12*** | -.00 | .49*** | — |  |  |  |  |  |  |  |  |  |  |
| 11. G_geom | -.04 | -.09** | -.19*** | -.00 | -.01 | -.03 | -.13*** | .00 | .45*** | .81*** | — |  |  |  |  |  |  |  |  |  |
| 12. G_eng | -.09** | -.15*** | -.07* | -.09** | -.04 | -.02 | -.14*** | -.05 | .39*** | .37*** | .40*** | — |  |  |  |  |  |  |  |  |
| 13. G_lit | -0.043 | -.05 | -.05 | -.01 | -.00 | -.02 | -.06 | -.00 | .40*** | .26*** | .33*** | .34*** | — |  |  |  |  |  |  |  |
| 14. G_inf | -0.055 | -.08* | -.08* | -.03 | -.01 | -.00 | -.08* | -.05 | .24*** | .32*** | .34*** | .21*** | .17*** | — |  |  |  |  |  |  |
| 15. G_hist | -.01 | -.11** | -.01 | -.03 | -.04 | -.02 | -.07* | .01 | .43*** | .37*** | .41*** | .39*** | .40*** | .28*** | — |  |  |  |  |  |
| 16. G_geog | -.02 | -.08* | -.02 | -.01 | -.00 | -.01 | -.11** | .02 | .36*** | .30*** | .34*** | .33*** | .32*** | .26*** | .51*** | — |  |  |  |  |
| 17. G_bio | -.03 | -.03 | -.01 | .01 | .02 | -.00 | -.05 | .03 | .34*** | .32*** | .41*** | .36*** | .30*** | .27*** | .40*** | .39*** | — |  |  |  |
| 18. G_soc | -.04 | -.10** | -.02 | -.05 | -.03 | -.03 | -.07* | -.02 | .38*** | .30*** | .35*** | .35*** | .36*** | .28*** | .59*** | .43*** | .37*** | — |  |  |
| 19. G_phy | -.05 | -.07* | -.06 | -.02 | -.01 | -.04 | -.09* | -.02 | .41*** | .50*** | .52*** | .40*** | .28*** | .33*** | .40*** | .37*** | .35*** | .43*** | — |  |
| 20. G_che | -.03 | -.05 | -.07* | .00 | .01 | -.03 | -.11** | .05 | .36*** | .42*** | .49*** | .32*** | .26*** | .20*** | .35*** | .32*** | .44*** | .39*** | .50*** | — |
| 21. G_ast | .09 | .13 | .11 | .12 | .06 | .12 | -.19* | .12 | .34*** | .21** | .31*** | .52*** | .30*** | .36*** | .27*** | .38*** | .49*** | .31*** | .38*** | .56*** |

Note: *STAIT – State Trait Anxiety Inventory – Trait subscale; State Trait Anxiety Inventory – State subscale; AMAS – Abbreviated Maths Anxiety Scale; GAD - Generalized Anxiety Disorder Questionnaire; ASC – Appraisal of Social Concerns; CASI – Childhood Anxiety Scale; SAnx – Spatial Anxiety; PSWQ – Penn State Worry Questionnaire; G_rus – Russian Language Grade; G_alg – Algebra Grade; G_gem – Geometry Grade; G-eng – English language Grade; G_lit – Literature Grade; G_inf – Informatics Grade; G_hist – History Grade; G_geog – Geography Grade; G_bio – Biology Grade; G-soc – Sociology Grade; G_phy – Physics Grade; G_che – Chemistry Grade; G_ast – Astronomy Grade;*** p<.001; **p<.01, *p<.05*

# **Table S5. Gender and expertise differences in 13 subject grades measures**

|  | Effects | | | | | |
| --- | --- | --- | --- | --- | --- | --- |
|  | Gender | | Expertise | | interaction | |
|  | eta^2 | sig | eta^2 | sig | eta^2 | sig |
| G_rus | .02 | <.001 | .09 | <.001 | .01 | ns |
| G_alg | .00 | ns | .11 | <.001 | .00 | ns |
| G_geom | .00 | ns | .11 | <.001 | .00 | ns |
| G_eng | .01 | <.001 | .06 | <.001 | .00 | ns |
| G_lit | .02 | <.001 | .03 | <.001 | .00 | ns |
| G_inf | .01 | <.001 | .02 | <.001 | .01 | <.01 |
| G_hist | .01 | .001 | .05 | <.001 | .00 | ns |
| G_geog | .01 | <.05 | .05 | <.001 | .00 | ns |
| G_bio | .01 | <.001 | .05 | <.001 | .00 | ns |
| G_soc | .00 | ns | .04 | <.001 | .00 | ns |
| G_phy | .00 | ns | .09 | <.001 | .00 | ns |
| G_che | .00 | ns | .13 | <.001 | .00 | ns |
| G_ast* | .09 | <.001 | NA | NA | NA | NA |
| Note: due to missing data an interaction term between Gender and Expertise was not computed; and the effect size is larger probably because the expertise variable is not accounted for in the model | | | | | | |

# **Figure S1 Heatmap for correlations among 8 anxiety measures and 13 school grades in Science experts**


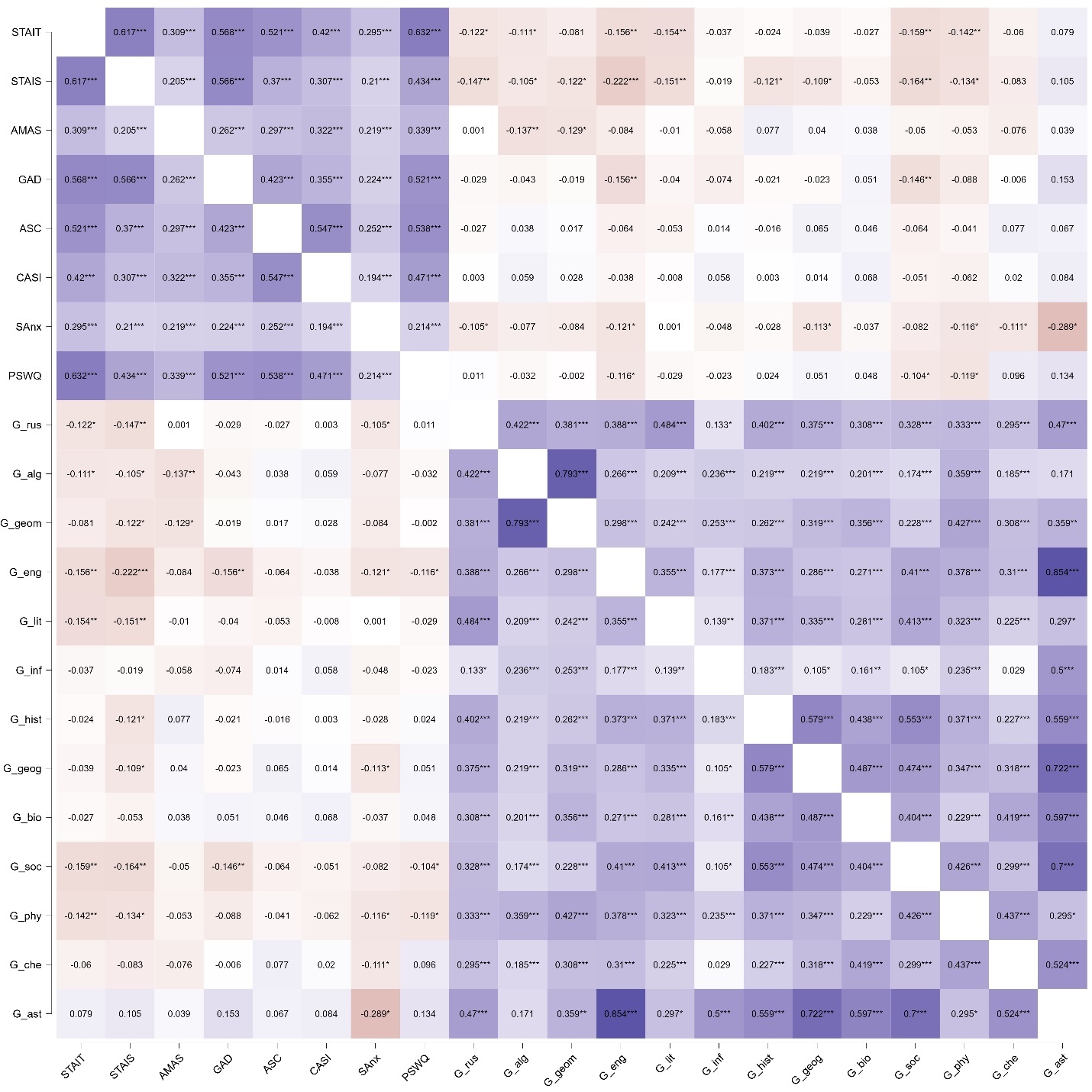


Note: *STAIT – State Trait Anxiety Inventory – Trait subscale; State Trait Anxiety Inventory – State subscale; AMAS – Abbreviated Maths Anxiety Scale; GAD - Generalized Anxiety Disorder Questionnaire; ASC – Appraisal of Social Concerns; CASI – Childhood Anxiety Scale; SAnx – Spatial Anxiety; PSWQ – Penn State Worry Questionnaire; G_rus – Russian Language Grade; G_alg – Algebra Grade; G_gem – Geometry Grade; G-eng – English language Grade; G_lit – Literature Grade; G_inf – Informatics Grade; G_hist – History Grade; G_geog – Geography Grade; G_bio – Biology Grade; G-soc – Sociology Grade; G_phy – Physics Grade; G_che – Chemistry Grade; G_ast – Astronomy Grade;*** p<.001; **p<.01, *p<.05*

# **Figure S2 Heatmap for correlations among 8 anxiety measures and 13 school grades in Arts experts**


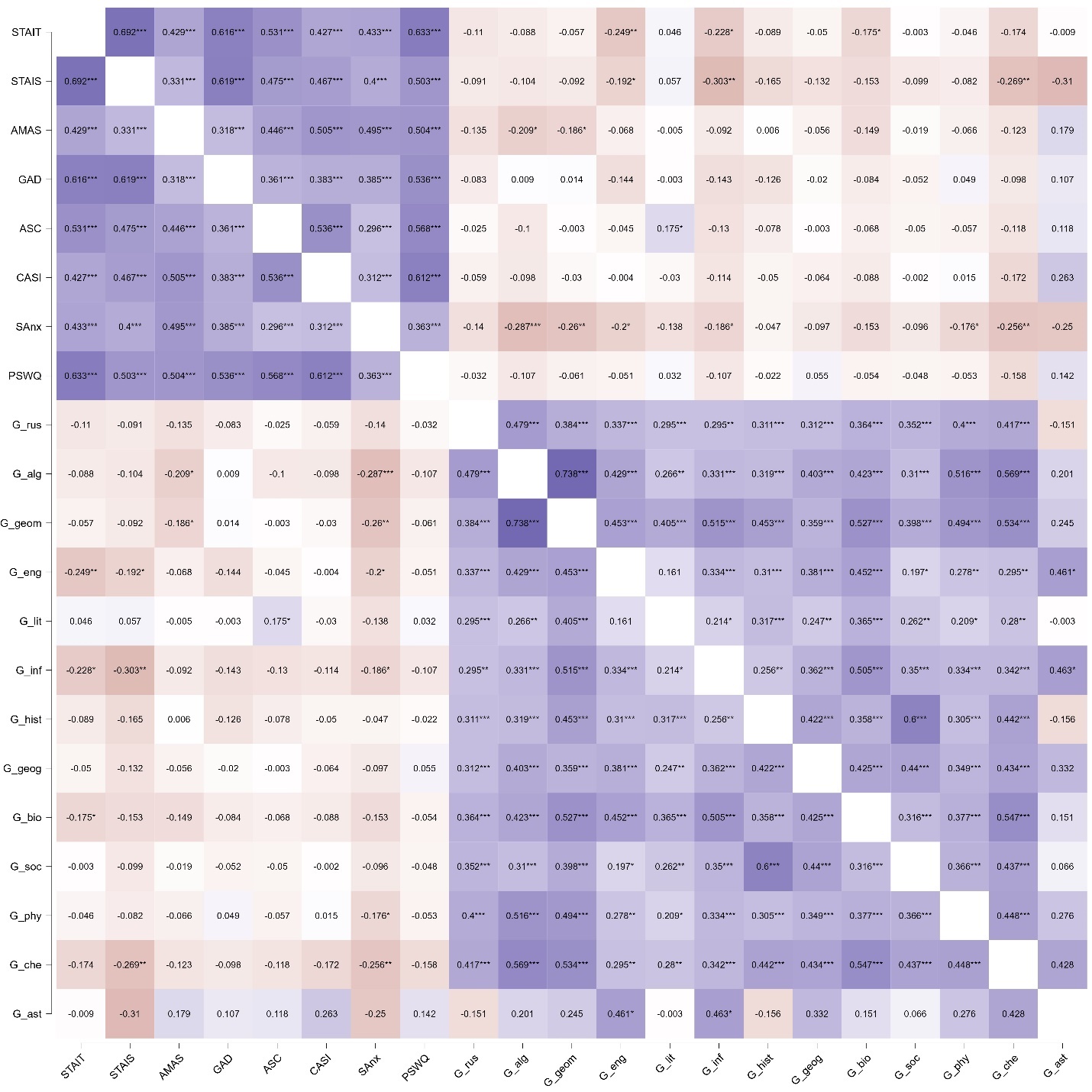


Note: *STAIT – State Trait Anxiety Inventory – Trait subscale; State Trait Anxiety Inventory – State subscale; AMAS – Abbreviated Maths Anxiety Scale; GAD - Generalized Anxiety Disorder Questionnaire; ASC – Appraisal of Social Concerns; CASI – Childhood Anxiety Scale; SAnx – Spatial Anxiety; PSWQ – Penn State Worry Questionnaire; G_rus – Russian Language Grade; G_alg – Algebra Grade; G_gem – Geometry Grade; G-eng – English language Grade; G_lit – Literature Grade; G_inf – Informatics Grade; G_hist – History Grade; G_geog – Geography Grade; G_bio – Biology Grade; G-soc – Sociology Grade; G_phy – Physics Grade; G_che – Chemistry Grade; G_ast – Astronomy Grade;*** p<.001; **p<.01, *p<.05*

# **Figure S3 Heatmap for correlations among 8 anxiety measures and 13 school grades in Sports experts**


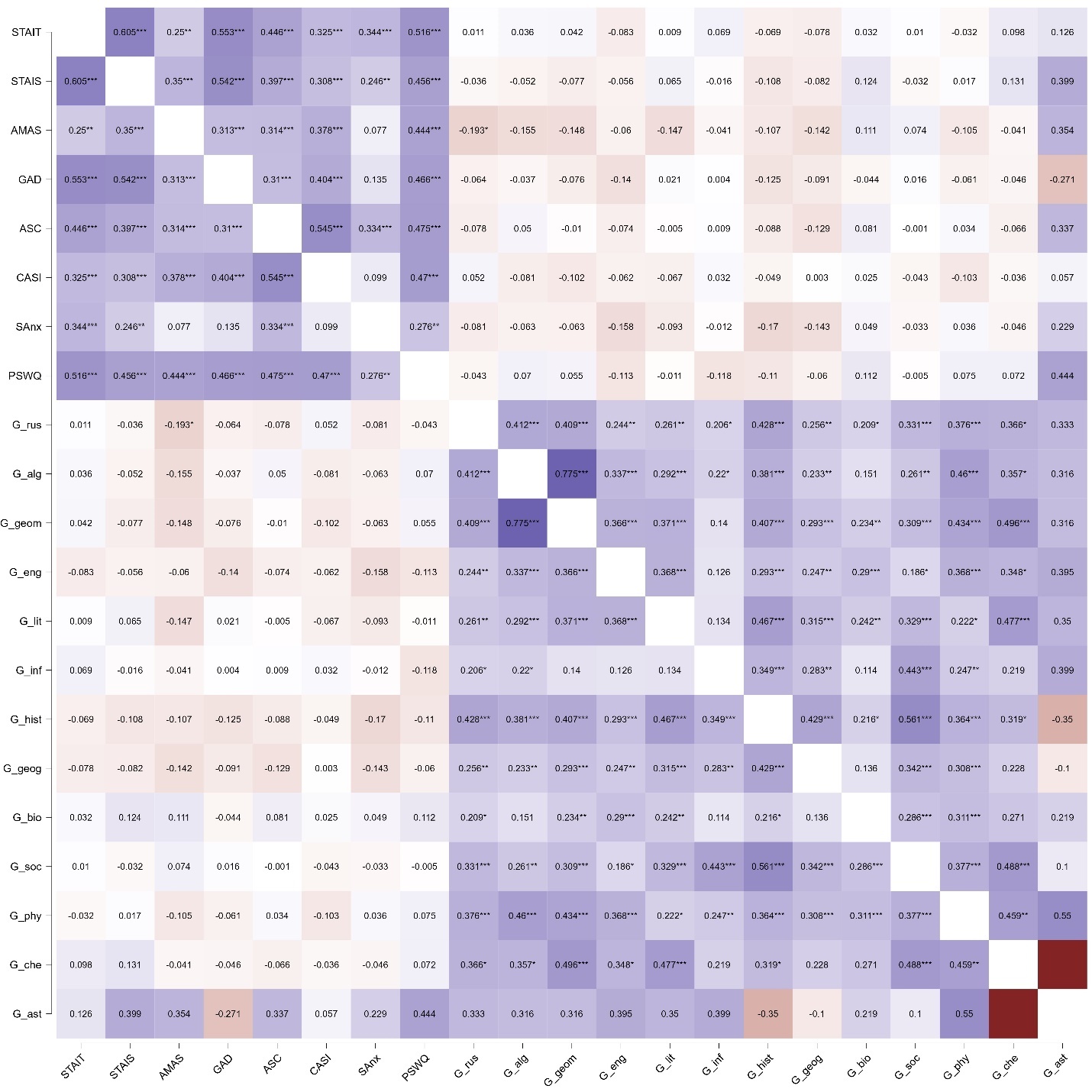


Note: *STAIT – State Trait Anxiety Inventory – Trait subscale; State Trait Anxiety Inventory – State subscale; AMAS – Abbreviated Maths Anxiety Scale; GAD - Generalized Anxiety Disorder Questionnaire; ASC – Appraisal of Social Concerns; CASI – Childhood Anxiety Scale; SAnx – Spatial Anxiety; PSWQ – Penn State Worry Questionnaire; G_rus – Russian Language Grade; G_alg – Algebra Grade; G_gem – Geometry Grade; G-eng – English language Grade; G_lit – Literature Grade; G_inf – Informatics Grade; G_hist – History Grade; G_geog – Geography Grade; G_bio – Biology Grade; G-soc – Sociology Grade; G_phy – Physics Grade; G_che – Chemistry Grade; G_ast – Astronomy Grade;*** p<.001; **p<.01, *p<.05*

# **Figure S4 Heatmap for correations among 8 anxiety measures and 13 school grades in Literature experts**


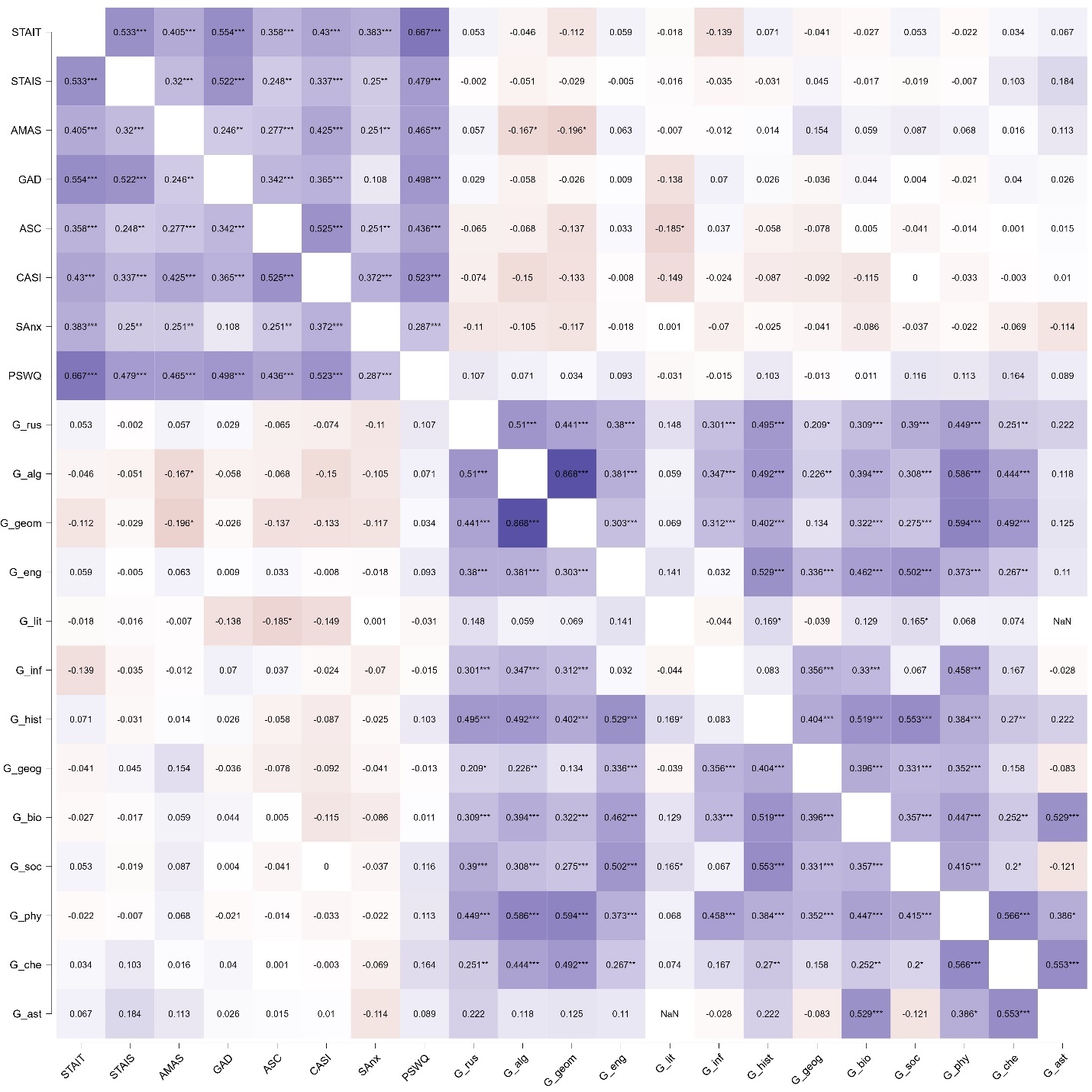


Note: *STAIT – State Trait Anxiety Inventory – Trait subscale; State Trait Anxiety Inventory – State subscale; AMAS – Abbreviated Maths Anxiety Scale; GAD - Generalized Anxiety Disorder Questionnaire; ASC – Appraisal of Social Concerns; CASI – Childhood Anxiety Scale; SAnx – Spatial Anxiety; PSWQ – Penn State Worry Questionnaire; G_rus – Russian Language Grade; G_alg – Algebra Grade; G_gem – Geometry Grade; G-eng – English language Grade; G_lit – Literature Grade; G_inf – Informatics Grade; G_hist – History Grade; G_geog – Geography Grade; G_bio – Biology Grade; G-soc – Sociology Grade; G_phy – Physics Grade; G_che – Chemistry Grade; G_ast – Astronomy Grade;*** p<.001; **p<.01, *p<.05*

# **Table S6 Factor loadings for “General vs. Specific” CFA model**

| *Factor Loadings* | | | | | | | | | | | | | | | | | | | | | | | |
| --- | --- | --- | --- | --- | --- | --- | --- | --- | --- | --- | --- | --- | --- | --- | --- | --- | --- | --- | --- | --- | --- | --- | --- |
|  | | | | | | | | | | | | | | 95% Confidence Interval | | | | Standardized | | | | | |
| Latent | | Indicator | |  | | Estimate | | Std. Error | | z-value | | p | | Lower | | Upper | | All | | LV | | Endo | |
| Gen |  | STAIT |  |  |  | 1.00 |  | 0.00 |  |  |  |  |  | 1.00 |  | 1.00 |  | 0.85 |  | 7.73 |  | 0.85 |  |
|  |  | STAIS |  |  |  | 0.77 |  | 0.04 |  | 21.31 |  | < .001 |  | 0.70 |  | 0.85 |  | 0.71 |  | 5.98 |  | 0.71 |  |
|  |  | GAD |  |  |  | 0.30 |  | 0.01 |  | 22.20 |  | < .001 |  | 0.27 |  | 0.33 |  | 0.73 |  | 2.31 |  | 0.73 |  |
|  |  | PSWQ |  |  |  | 1.12 |  | 0.04 |  | 24.94 |  | < .001 |  | 1.03 |  | 1.20 |  | 0.80 |  | 8.62 |  | 0.80 |  |
| Spec |  | SAnx |  |  |  | 1.00 |  | 0.00 |  |  |  |  |  | 1.00 |  | 1.00 |  | 0.40 |  | 0.40 |  | 0.40 |  |
|  |  | AMAS |  |  |  | 7.33 |  | 0.80 |  | 9.15 |  | < .001 |  | 5.76 |  | 8.90 |  | 0.56 |  | 2.94 |  | 0.56 |  |
|  |  | CASI |  |  |  | 10.72 |  | 1.07 |  | 10.05 |  | < .001 |  | 8.63 |  | 12.82 |  | 0.73 |  | 4.30 |  | 0.73 |  |
|  |  | ASC |  |  |  | 34.08 |  | 3.37 |  | 10.12 |  | < .001 |  | 27.48 |  | 40.68 |  | 0.75 |  | 13.67 |  | 0.75 |  |
|  | | | | | | | | | | | | | | | | | | | | | | | |
